# Unified Synthesis and Biological Evaluation of Makaluvamine J and Its Analogs

**DOI:** 10.3390/molecules29061389

**Published:** 2024-03-20

**Authors:** Yo Kiichi, Koshiro Fukuoka, Anna Kitano, Koya Ishino, Naoyuki Kotoku

**Affiliations:** College of Pharmaceutical Sciences, Ritsumeikan University, 1-1-1 Noji-higashi, Kusatsu, Shiga 525-8577, Japan; ph0111pv@ed.ritsumei.ac.jp (Y.K.); ph0142vx@ed.ritsumei.ac.jp (K.F.); ph0167vi@ed.ritsumei.ac.jp (A.K.); ph0128pr@ed.ritsumei.ac.jp (K.I.)

**Keywords:** Makaluvamine J, pancreatic cancer, analog synthesis, structure-activity relationship

## Abstract

Makaluvamine J, a pyrroloiminoquinone alkaloid of marine sponge origin, and its analogs were synthesized and assessed for their potential to develop as a novel and selective growth inhibitor targeting human pancreatic cancer PANC-1 cells. Ts-damirone B, a common precursor featuring a pyrroloiminoquinone core structure, was synthesized through Bartoli indole synthesis and IBX-mediated oxidation. Late-stage diversification at *N*-5 and *N*-9 yielded makaluvamine J and several analogs. A structure–activity relationship (SAR) analysis highlighted the significance of the lipophilic side chain at *N*-9 for the growth inhibitory activity of PANC-1 cells. The modest alkyl group at *N*-5 was found to improve selectivity against other cancer cells. Among the prepared analogs, the tryptamine analog **24** showed potent and selective cytotoxicity (IC_50_ = 0.029 µM, selective index = 13.1), exceeding those of natural products.

## 1. Introduction

Marine natural products are considered rich and promising sources of drug candidates in the field of anticancer drug discovery [1,2]. Our team has concentrated on discovering novel antitumor compounds and previously developed a screening system to identify selective growth inhibitors of cancer cells under glucose-deprived conditions from marine organisms and marine-derived microorganisms [3]. For instance, novel chlorinated polyketides designated as biakamides A–D were isolated as potent and selective growth inhibitors of human pancreatic cancer PANC-1 cells under glucose-deprived conditions from the extract of marine sponge *Petrosaspongia* sp. Mechanistic studies have uncovered that biakamides inhibit the mitochondrial respiratory chain complex I, leading to reduced ATP synthesis in PANC-1 cells [4,5]. Many other compounds exhibiting similar bioactivity, so-called “anti-austerity agents”, have been isolated through similar bioassay-guided separation [6,7,8,9,10]. Of these, GBS-01, an extract of *A. lappa* containing high levels of arctigenin*,* has undergone testing in a phase I clinical trial for patients with advanced pancreatic cancer resistant to gemcitabine [11]. This example indicates the efficacy of natural products in the development of new drug candidates against pancreatic cancer.

**Figure 1:**
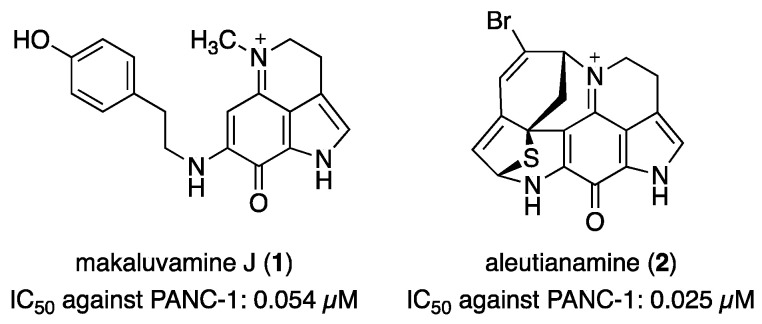
The chemical structures of makaluvamine J (**1**) and aleutianamine (**2**).

Clinically, pancreatic cancer is one of the most difficult cancers to treat, and no effective chemotherapeutic agents exist [12]. Therefore, the discovery of potent and selective growth inhibitors for pancreatic cancer cells is ongoing. Some pyrroloiminoquinone alkaloids are known to exhibit potent cytotoxicity against PANC-1 cells (Figure 1). Makaluvamine J (**1**) is one of the most potent active substances, with an IC_50_ value of 0.054 µM against PANC-1 cells [13]. Furthermore, aleutianamine (**2**), a novel heptacyclic pyrroloiminoquinone alkaloid obtained from an Antarctic marine sponge, demonstrated selective cytotoxicity against PANC-1 cells (IC_50_ = 0.025 µM) [14]. The significance of the compounds is well demonstrated by comparing them with the potency of gemcitabine (IC_50_ = 4 µM), a first-line chemotherapeutic agent approved by the FDA [15].

These findings suggest that pyrroloiminoquinone with cationic nitrogen at *N*-5 could be a crucial scaffold for demonstrating potent cytotoxicity against pancreatic cancer cells. To investigate this hypothesis and develop more potent compounds as potential anti-pancreatic cancer drug candidates, we synthesized makaluvamines and their analogs. This report details the synthesis and evaluation of makaluvamine J (**1**) and its analogs at *N*-5 and *C*-7.

## 2. Results and Discussions

### 2.1. Reported Bioactivity and SAR of Makaluvamines against Pancreatic Cancer Cells

**Figure 2:**
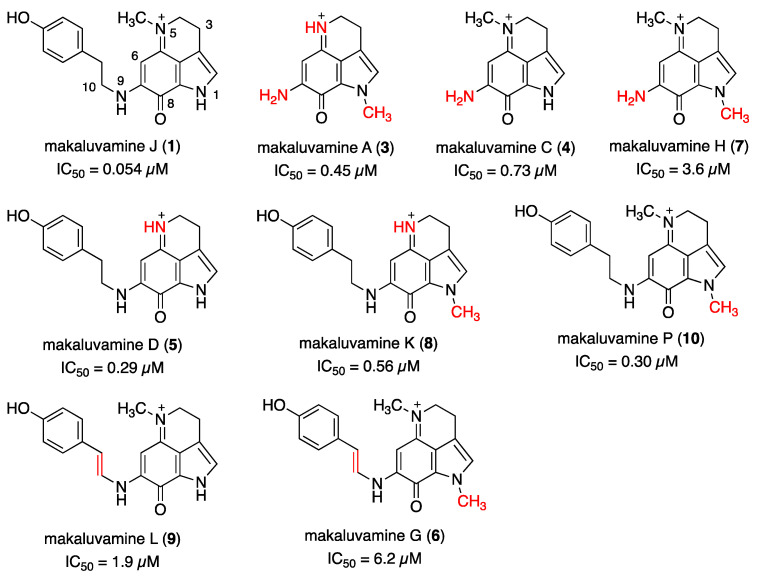
The structures and bioactivities of makaluvamines (**1**, **3**–**10**). Reported IC_50_ values of the respective compounds against PANC-1 cells are indicated. The structural differences from makaluvamine J (**1**) are highlighted in red.

The reported structure–cytotoxicity relationship of makaluvamines in PANC-1 cells is outlined in Figure 2 [13]. The key structural feature is the substituent at *N*-9: makaluvamines A (**3**), C (**4**), and H (**7**), which lack a substituent, displayed less than one-eighth the cytotoxicity of **1** against PANC-1; meanwhile, makaluvamines G (**6**) and L (**9**) with E-alkenyl side chains exhibited relatively lower potency. Given these results and the potent cytotoxicity of aleutianamine (**2**), an appropriately oriented substituent at *N*-9 appears crucial. Conversely, demethylation of *N*-5 (makaluvamine D (**5**)) or methylation of *N*-1 (makaluvamine P (**10**)) led to approximately one-fifth less potency than compound **1**, underscoring the significance of the substitution pattern at these positions.

### 2.2. Unified Divergent Synthesis of Makaluvamines and Analogs

**Scheme 1:**
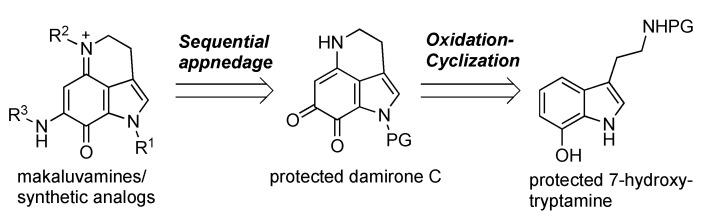
Outline of our synthetic approach toward makaluvamine derivatives.

There have been a number of reports on the total synthesis of makaluvamines [16,17,18,19,20,21,22], although most of them have focused on the development of synthetic methods for the characteristic tricyclic skeleton. To gather more detailed SAR information, a unified, divergent synthesis of natural makaluvamines or various analogs and their biological evaluations is required. Scheme 1 shows an outline of our approach toward these molecules. Protected damirone C served as a common precursor, with various substituents at *N*-1, *N*-5, and *N*-9 appended at the final stage of the synthesis. The damirone skeleton was prepared from a 7-hydroxytryptamine derivative via oxidation/cyclization.

**Scheme 2:**
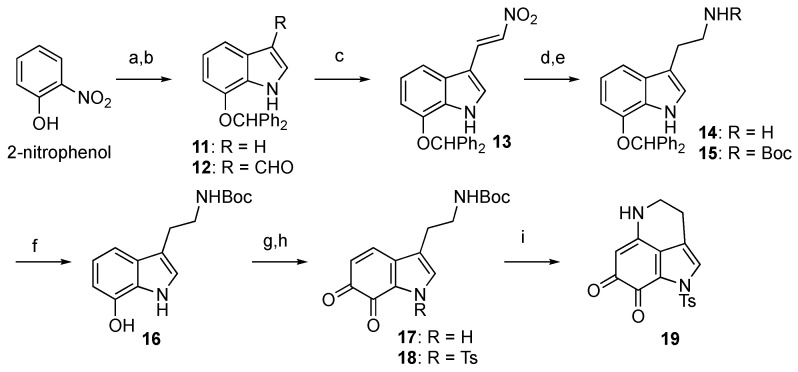
Synthesis of Ts-damirone C (**19**). Reagents and conditions: (a) Benzhydryl bromide, K_2_CO_3_, acetone, reflux; vinylmagnesium chloride, THF, –40 °C; (b) POCl_3_, DMF, 0 °C to rt, then KOH, H_2_O, 50 °C; (c) NH_4_OAc, CH_3_NO_2_, reflux, 86% (2 steps); (d) LiAlH_4_, THF, reflux; (e) (Boc)_2_O, Et_3_N, 1,4-dioxane, 55% (2 steps); (f) H_2_, Pd(OH)_2_-C, MeOH, 97%; (g) IBX, DMF, 0 °C; (h) TsCl, DMAP, Et_3_N, THF, 63% (2 steps); (i) TFA, CH_2_Cl_2_, 0 °C; Et_3_N, MeOH, 0 °C, 55%.

First, protected damirone C was synthesized as depicted in Scheme 2. As reported in the literature, 7-(benzhydryloxy)indole (**11**) was synthesized using Bartoli indole synthesis, commencing with 2-nitrophenol [23]. Then, **11** was converted into tryptamine derivative **16** as follows: regioselective introduction of the side chain was achieved through Vilsmeier–Haack formylation and a subsequent Henry reaction to give nitroalkene **13** in good yield. The reduction of nitroalkene **13** with LiAlH_4_ yielded amine **14**, which was isolated as Boc carbamate **15** in moderate yield. Hydrogenolysis of the benzhydryl ether of **15** gave 7-hydroxytryptamine derivative **16**, and subsequent oxidation to the corresponding *ortho*-quinone **17** was attempted. The use of 2-iodoxybenzoic acid (IBX) in *N*,*N*-dimethylformamide (DMF) [24,25] was found to be effective, although the reaction yield was moderate (<50%) with commercially available IBX (purchased from TCI Chemicals, >39% purity). However, when lab-made pure IBX was used, better results were obtained, with an isolated yield of 63% after the protection of the pyrrole nitrogen as *p*-toluenesulfonamide **18**. The final steps involved intramolecular cyclization by removing the Boc group with TFA and subsequent Et_3_N treatment to yield the desired Ts-damirone C (**19**) in moderate yield.

**Scheme 3:**
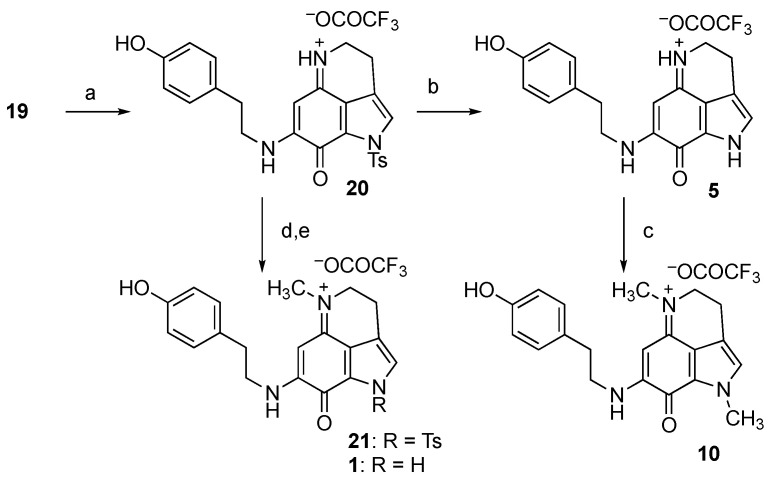
Attempted synthesis of makaluvamines. Reagents and conditions: (a) Tyramine, Et_3_N, MeOH, then TFA, 49%, 87% brsm; (b) pyridine hydrochloride, DMF, 80 °C, 70%; (c) MeI, NaH, DMF, 92%; (d) MeI, NaH, DMF; (e) pyridine hydrochloride, DMF, 80 °C.

With the common precursor **19** in hand, the next step involved attempting to append the substituents at *N*-1, *N*-5, and *N*-9 for the total synthesis of makaluvamines, as shown in Scheme 3. The condensation with tyramine under basic conditions afforded Ts-makaluvamine D (**20**) in 49% yield. A total of 38% of the unreacted **19** was also isolated in this case, indicating that the yield based on the recovered starting material (brsm) was 87%. The various orders of transformation of *N*-1 and *N*-5 were then examined. In the first attempt, the Ts-protecting group was removed, followed by *N*-methylation to prepare makaluvamine P (**10**). Pyridine hydrochloride in DMF [26] was identified as the optimal reagent for removing the Ts group, yielding makaluvamine D (**5**) in a good yield. Other attempted detosylation conditions (Cs_2_CO_3_ in THF/MeOH [27], NaH in DMF [28], and TFA/Me_2_S [29]) were not effective. Double *N*-methylation at *N*-1 and *N*-5 occurred following treatment with iodomethane in the presence of NaH, resulting in the synthesis of makaluvamine P (**10**) in excellent yield.

The preparation of makaluvamine J (**1**) was attempted through *N*-methylation at *N*-5 and the subsequent removal of the Ts group, in reverse order under similar conditions as described earlier. However, the MS and ^1^H NMR spectra of the crude product indicated that the starting material was decomposed, and a mixture of unidentified compounds was obtained instead of **1**.

**Scheme 4:**
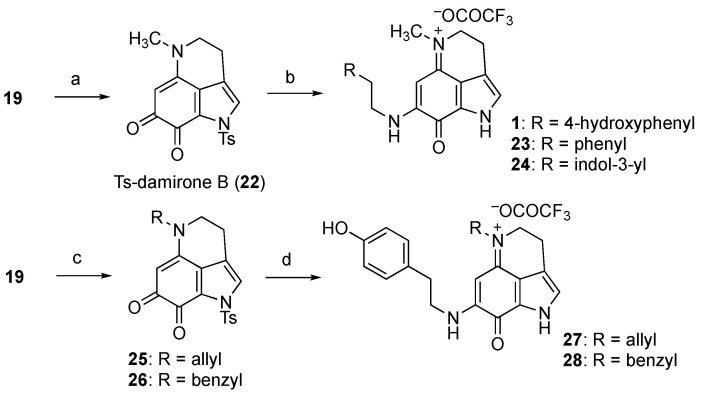
Synthesis of makaluvamine J (**1**) and analogs **23**–**28**. Reagents and conditions: (a) MeI, K_2_CO_3_, DMF, quant.; (b) RCH_2_CH_2_NH_2_, Et_3_N, MeOH, 37%, 87% brsm for **1**; 36%, 83% brsm for **23**; 22%, 74% brsm for **24**; (c) R-Br, K_2_CO_3_, DMF, 93% for **25**; 77% for **26**; (d) Tyramine, Et_3_N, MeOH, 22%, 89% brsm for **27**; 17%, 76% brsm for **28**.

Considering these findings, we examined another synthetic route, as depicted in Scheme 4. Thus, the methylation of *N*-5 of **19** gave Ts-damirone B (**22**) in quantitative yield. Unexpectedly, the subsequent treatment of **22** with tyramine (three equiv.) yielded makaluvamine J (**1**) in its pure form. It appears that condensation with ketone at *C*-7 and deprotection of the Ts group at *N*-1 occurred simultaneously, suggesting that this order of the appendage might be optimal for synthesis. Indeed, the use of phenethylamine or tryptamine instead of tyramine provided the corresponding unnatural makaluvamine analogs **23** and **24**, respectively. 

Furthermore, we similarly obtained the *N*-5 analogs. Thus, **19** was treated with allyl bromide or benzyl bromide to give the corresponding damirone analogs **25** and **26**, respectively. Subsequent condensation with tyramine and removal of the Ts group provided **27** and **28**, which could be used to analyze the participation of the *N*-5 substituent of makaluvamines in cytotoxicity against pancreatic cancer cells. 

**Figure 3:**
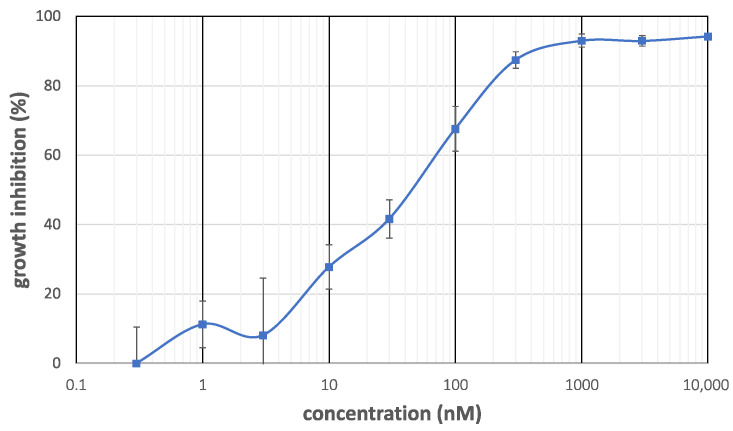
Growth inhibition curve of makaluvamine J (**1**) against PANC-1 cells.

The cytotoxicity of makaluvamine J (**1**) and analogs **23–28** against PANC-1 cells and human epidermoid carcinoma KB3-1 cells was evaluated as summarized in Table 1. The selective index, the ratio of potency between these two cell lines, is also shown in Table 1 to clearly depict the selectivity. The growth inhibition curve of **1** against PANC-1 cells (Figure 3) shows that the cytotoxicity was raised in an almost linear and dose-dependent manner in the range between 3 and 300 nM, and the concentration of 50% inhibition (IC_50_) was determined to be 0.046 µM. It was almost comparable to the reported one [13] (IC_50_ = 0.054 µM), which ensured the validity of our experiment. It was also found that **1** exhibited relatively weak cytotoxicity against KB3-1 cells (IC_50_ = 0.2 µM), with a selective index of 4.3. Weaker cytotoxicity and lower cell selectivity of makaluvamines D (**5**) and P (**10**) also supported the reported SAR, the necessity of quaternary nitrogen at *N*-5, and the negative effect of *N*-1 alkylation. The makaluvamine analogs prepared in this work (**23**, **24**, **27**, and **28**) also showed similar cytotoxicity against PANC-1 cells (0.027 to 0.042 µM), whereas significantly weakened cytotoxicity was observed with two damirone analogs (**25** and **26)**. In addition, cytotoxicity testing against KB3-1 cells revealed an intriguing relationship between structure and cytotoxicity. Thus, *N*-9 analogs **23** and **24** showed 10-fold lower cytotoxicity in KB3-1 cells, whereas *N*-5 analogs **27** and **28** exhibited comparable activity. This indicates that the *N*-5 substituent might contribute to cancer cell selectivity, and the *N*-9 substituent could enhance nonselective cytotoxicity. 

What surprised us the most was the potency of the analogs **23** and **24** against PANC-1 cells. The reported SAR showed that the 4-ethylphenol substituent is important for exhibiting potency against PANC-1 cells. On the other hand, analogs **23** and **24** possess 2-phenethyl and 2-(1*H*-indol-3-yl)ethyl moieties, respectively, instead of the 4-ethylphenol group. This indicates that the hydroxyl group at the side chain terminal is not necessary for exhibiting potent cytotoxicity. Although further studies are needed, the findings in this work are significant for optimizing the structural motif in this part.

In summary, the synthesis and biological evaluation of makaluvamine J (**1**) and its analogs revealed the importance of substituents *N*-5 and *N*-9 for cytotoxicity and pancreatic cancer cell selectivity. The unified synthetic method used in this study enabled easy access to makaluvamines and various analogs, yielding tryptamine analog **24** with more potent cytotoxicity against PANC-1 cells and enhanced selectivity over KB3-1 cells. Further synthesis and evaluation of various analogs will lead to the development of optimized anti-pancreatic cancer drug candidates. In addition, a mechanistic study of the compound, which will be undertaken in due course, will allow us to identify key molecules for overcoming pancreatic cancer.

## 3. Materials and Methods

### 3.1. General

The following instruments were used to obtain physical data: a JEOL JNM-ECZ500R/S1 (^1^H-NMR: 500 MHz, ^13^C-NMR: 125 MHz) spectrometer for ^1^H and ^13^C NMR data using tetramethylsilane as an internal standard; a Waters Xevo G2-XS Q-Tof mass spectrometer for ESI-Q-TOF MS. Silica gel (Kanto 63–210 μm) and pre-coated thin-layer chromatography (TLC) plates (Merck 60F_254_) were used for column chromatography and TLC. The spots on the TLC plates were detected by spraying with an acidic *p*-anisaldehyde solution (*p*-anisaldehyde: 25 mL, *c*-H_2_SO_4_: 25 mL, AcOH: 5 mL, EtOH: 425 mL) with subsequent heating. Unless otherwise noted, all reactions were performed under a N_2_ atmosphere. Hard copies of NMR spectra can be found as Supplementary Materials.

### 3.2. Antiproliferative Activity of the Compounds against Cancer Cells

PANC-1 and KB3-1 cells were cultured in RPMI-1640 medium supplemented with 10% heat-inactivated fetal bovine serum and kanamycin (50 μg/mL). Cells were plated into 96-well microplates at 5 × 10^3^ cells/100 μL assay medium/well, and various concentrations of test compounds were added to each well as a 10% DMSO/EtOH solution (1 µL). The plates were incubated at 37 °C in a humidified atmosphere of 5% CO_2_ for 72 h, and cell proliferation was determined by MTT colorimetric assay. IC_50_ values, drug concentrations that produced 50% cell death, were determined by interpolation of the plotted data (the averaged values of the triplicate experiment).

### 3.3. Synthesis

#### 3.3.1. 7-(Benzhydryloxy)-1*H*-indole (**11**)

K_2_CO_3_ (30.3 g, 219 mmol, 1.6 equiv.) and benzhydryl bromide (33.9 g, 137 mmol, 1.0 equiv.) were added to a solution of 2-nitrophenol (19.0 g, 137 mmol) in acetone (350 mL), and the red mixture was stirred with reflux for 6 h. After cooling to rt, the reaction mixture was filtered and concentrated in vacuo. The residue was extracted with Et_2_O, and the precipitates were removed by filtration. The filtrate was again concentrated in vacuo, and the residue was triturated with hexane (80 °C). The resulting brown solid was isolated by filtration, washed with hexane, and dried in vacuo to afford 2-benzhydryloxynitrobenzene (29.1 g, 70%) as a brown solid.

Vinylmagnesium chloride solution (1.4 mol/L in THF, 100 mL, 140 mmol, 3.5 equiv.) was added dropwise over a few minutes to a solution of an aliquot of the above product (12.2 g, 40.0 mmol) in dry THF (500 mL) at −40 °C, and the mixture was stirred for 45 min. Then, the reaction mixture was quenched with sat. NH_4_Cl aq. and the whole mixture was extracted with Et_2_O three times. The combined organic layer was dried over Na_2_SO_4_ and was concentrated in vacuo. The crude brown oil was purified by silica gel column chromatography (hexane/EtOAc = 7:1) to give **11** (6.0 g, 50%) as a pale yellow solid.

All the spectral data were identical to the reported ones [23].

#### 3.3.2. 7-(Benzhydryloxy)-1*H*-indole-3-carbaldehyde (**12**)

POCl_3_ (2.00 mL, 22.0 mmol, 1.1 equiv.) was added dropwise to dry DMF (20 mL) at 0 °C, and a solution of **11** (6.01 g, 20.0 mmol) in dry DMF (10 mL) was added dropwise to the reaction mixture. The resulting yellow mixture was allowed to warm up to rt with stirring for 3 h. After cooling to 0 °C, a solution of KOH (6.80 g) in H_2_O (30 mL) was added to the reaction mixture, and the whole mixture was stirred at 50 °C for 90 min. After cooling to rt, the resulting mixture was diluted with EtOAc, and the whole mixture was washed with H_2_O and brine. The organic layer was dried over Na_2_SO_4_ and was concentrated in vacuo to afford a crude **12** (5.98 g) as a brown oil. The residue was subjected to the next reaction without further purification.

A characterization sample could be obtained by silica gel column chromatography (hexane/EtOAc = 4:1) as a pale-yellow powder.

^1^H NMR (500 MHz, CDCl_3_): δ 9.97 (s, 1H), 9.56 (br s, 1H), 7.86 (d, *J* = 7.9 Hz, 1H), 7.73 (d, *J* = 2.9 Hz, 1H), 7.43 (d, *J* = 7.3 Hz, 4H), 7.34 (t, *J* = 7.4 Hz, 4H), 7.30 (t, *J* = 7.3 Hz, 2H), 7.08 (t, *J* = 8.0 Hz, 1H), 6.70 (d, *J* = 7.9 Hz, 1H), 6.40 (s, 1H). ^13^C NMR (125 MHz, CDCl_3_): δ 185.6, 144.6, 140.7, 135.1, 128.8, 128.2, 127.8, 127.1, 126.1, 123.7, 120.0, 114.7, 107.7, 82.5. MS (ESI-Q-TOF) *m/z*: 328 [M + H]^+^. HRMS (ESI-Q-TOF) *m/z*: 328.1338, calcd for C_22_H_18_NO_2_; found: 328.1354.

#### 3.3.3. (*E*)-7-(Benzhydryloxy)-3-(2-nitrovinyl)-1*H*-indole (**13**)

Ammonium acetate (1.23 g, 22.0 mmol, 1.1 equiv.) was added to a solution of crude **12** (5.98 g, 18.3 mmol) in nitromethane (50 mL), and the red mixture was stirred with reflux for 2 h. After cooling to rt, the reaction mixture was concentrated in vacuo. The residue was diluted with CH_2_Cl_2_, and the whole mixture was washed with H_2_O and brine. The organic layer was dried over Na_2_SO_4_ and was concentrated in vacuo to a red solid. Recrystallization from CH_2_Cl_2_ was performed to afford pure **13** (6.43 g, 86% over 2 steps) as an orange solid.

^1^H NMR (500 MHz, (CD_3_)_2_CO): δ 11.71 (br s, 1H), 8.37 (d, *J* = 13.4 Hz, 1H), 8.14 (d, *J* = 3.0 Hz, 1H), 7.87 (d, *J* = 13.4 Hz, 1H), 7.62 (d, *J* = 7.4 Hz, 4H), 7.48 (d, *J* = 8.0 Hz, 1H), 7.36 (t, *J* = 7.5 Hz, 4H), 7.27 (t, *J* = 7.4 Hz, 2H), 7.09 (t, *J* = 8.0 Hz, 1H), 6.91 (d, *J* = 8.0 Hz, 1H), 6.73 (1H, s). ^13^C NMR (125 MHz, (CD_3_)_2_CO): δ 145.7, 142.5, 135.1, 134.8, 132.9, 129.6, 129.4, 128.6, 127.6, 123.5, 114.0, 110.2, 108.0, 81.9. MS (ESI-Q-TOF) *m/z*: 371 [M + H]^+^. HRMS (ESI-Q-TOF) *m/z*: 371.1396, calcd for C_23_H_19_N_2_O_3_; found: 371.1391.

#### 3.3.4. 2-(7-(Benzhydryloxy)-1*H*-indol-3-yl)ethan-1-amine (**14**)

LiAlH_4_ (3.98 g, 104 mmol, 6.0 equiv.) was carefully added to a solution of **13** (6.43 g, 17.4 mmol) in dry THF (90 mL) at 0 °C, and the green mixture was stirred with reflux for 3 h. After cooling to 0 °C, the reaction mixture was quenched by slow addition of sat. Na_2_SO_4_ aq. (90 mL). The reaction mixture was passed through a pad of Celite and was washed with EtOAc. Brine was added to the filtrate and extracted with EtOAc twice. The organic layer was dried over Na_2_SO_4_ and was concentrated in vacuo to afford a crude **14** (5.43 g) as a pale yellow solid. The residue was subjected to the next reaction without further purification.

A characterization sample could be obtained by silica gel column chromatography (hexane/EtOAc = 4:1) as a white solid.

^1^H NMR (500 MHz, CDCl_3_): δ 9.04 (br s, 1H), 7.46 (d, *J* = 7.3 Hz, 4H), 7.37 (t, *J* = 7.3 Hz, 4H), 7.32 (t, *J* = 7.3 Hz, 2H), 7.21 (d, *J* = 7.9 Hz, 1H), 6.96 (s, 1H), 6.92 (t, *J* = 7.9 Hz, 1H), 6.59 (d, *J* = 7.7 Hz, 1H), 6.41 (s, 1H), 2.99 (t, *J* = 6.6 Hz, 2H), 2.88 (t, *J* = 6.6 Hz, 2H), 1.59 (br s, 2H). ^13^C NMR (125 MHz, CDCl_3_): δ 144.8, 141.2, 129.1, 128.7, 127.9, 127.5, 127.1, 121.9, 119.5, 113.9, 112.0, 105.0, 82.1, 42.3, 29.5. MS (ESI-Q-TOF) *m/z*: 343 [M + H]^+^. HRMS (ESI-Q-TOF) *m/z*: 343.1810, calcd for C_23_H_23_N_2_O; found: 343.1815.

#### 3.3.5. *tert*-Butyl (2-(7-(benzhydryloxy)-1*H*-indol-3-yl)ethyl)carbamate (**15**)

Et_3_N (5.51 mL, 39.5 mmol) and (Boc)_2_O (3.75 mL, 17.4 mmol) were added dropwise to a suspended solution of crude **14** (5.43 g) in 1,4-dioxane (30 mL), and the whole mixture was stirred for 30 min. The reaction mixture was concentrated in vacuo, and the crude product was purified by silica gel column chromatography (hexane/EtOAc = 6:1) to give **15** (4.23 g, 55% over 2 steps) as a pale yellow powder.

^1^H NMR (500 MHz, CDCl_3_): δ 8.40 (br s, 1H), 7.46 (d, *J* = 7.0 Hz, 4H), 7.38 (t, *J* = 7.5 Hz, 4H), 7.32 (t, *J* = 7.3 Hz, 2H), 7.21 (d, *J* = 7.9 Hz, 1H), 6.97 (s, 1H), 6.92 (t, *J* = 7.9 Hz, 1H), 6.60 (d, *J* = 7.9 Hz, 1H), 6.40 (s, 1H), 4.67 (br s, 1H), 3.46 (d, *J* = 6.6 Hz, 2H), 2.95 (t, *J* = 6.6 Hz, 2H), 1.48 (s, 9H). ^13^C NMR (125 MHz, CDCl_3_): δ 156.1, 144.8, 141.3, 129.0, 128.8, 128.0, 127.5, 127.1, 121.8, 119.8, 113.7, 112.0, 105.3, 82.2, 79.2, 40.9, 28.5, 26.0. MS (ESI-Q-TOF) *m/z*: 443 [M + H]^+^. HRMS (ESI-Q-TOF) *m/z*: 443.2335, calcd for C_28_H_31_N_2_O_3_; found: 443.2315.

#### 3.3.6. *tert*-Butyl (2-(7-hydroxy-1*H*-indol-3-yl)ethyl)carbamate (**16**)

Pd(OH)_2_-C (20% *w*/*w*, 0.20 g, 0.03 mmol, 0.03 equiv.) was added to a solution of **15** (4.23 g, 9.56 mmol) in MeOH (50 mL), and the mixture was stirred for 8 h under a H_2_ atmosphere. The reaction mixture was filtered through a pad of Celite and was concentrated in vacuo. The crude product was purified by silica gel column chromatography (hexane/EtOAc = 3:2) to give **16** (2.55 g, 97%) as a white powder.

The spectroscopic and physical data were identical to those reported [25].

#### 3.3.7. *tert*-Butyl (2-(6,7-dioxo-6,7-dihydro-1*H*-indol-3-yl)ethyl)carbamate (**17**)

IBX (2.84 g, 10.2 mmol, 1.1 equiv.) was added to a solution of **16** (2.55 g, 9.23 mmol) in dry DMF (50 mL) at 0 °C, and the mixture was stirred for 2 h. The red mixture was quenched with sat. NaHCO_3_ aq. and the whole mixture was extracted with EtOAc. The combined organic layer was washed with H_2_O, dried over Na_2_SO_4_, and concentrated in vacuo to afford crude **17** (2.23 g) as a brown solid. The residue was subjected to the next reaction without further purification.

A characterization sample could be obtained by silica gel column chromatography (hexane/EtOAc = 1:2) as an orange solid.

The spectroscopic and physical data were identical to those reported [25].

#### 3.3.8. *tert*-Butyl (2-(6,7-dioxo-1-tosyl-6,7-dihydro-1*H*-indol-3-yl)ethyl)carbamate (**18**)

Et_3_N (3.19 mL, 23.0 mmol) was added dropwise to a solution of crude **17** (2.23 g) in THF (150 mL) at 0 °C. Then, 4-dimethylaminopyridine (0.23 g, 1.92 mmol) and *p*-toluenesulfonyl chloride (3.66 g, 19.2 mmol) were successively added to the solution, and the whole mixture was stirred for 1 h. The reaction mixture was quenched with 0.2 mol/L HCl aq. and extracted with EtOAc three times. The organic layer was dried over Na_2_SO_4_ and was concentrated in vacuo. The crude material was purified by silica gel column chromatography (CH_2_Cl_2_/EtOAc = 15:1) to give **18** (2.58 g, 63% over 2 steps) as a red solid.

The spectroscopic and physical data were identical to those reported [25].

#### 3.3.9. 1-Tosyl-1,3,4,5-tetrahydropyrrolo[4,3,2-*de*]quinoline-7,8-dione (Ts-damirone C, **19**)

TFA (11.5 mL, 150 mmol, 50 equiv.) was added to a solution of **18** (1.34 g, 3.00 mmol) in CH_2_Cl_2_ (50 mL) over 10 min at 0 °C, and the mixture was stirred for 70 min. The reaction mixture was concentrated in vacuo, and the resulting red-brown oil was redissolved in MeOH (60 mL). Et_3_N (4.16 mL, 30 mmol, 10.0 equiv.) was added to a solution over 10 min at 0 °C, and the mixture was stirred for 20 min. The reaction mixture was concentrated in vacuo, and the residue was diluted with CH_2_Cl_2_ and H_2_O. The layers were separated, and the aqueous layer was reextracted with CH_2_Cl_2_ twice. The combined organic layer was washed, saturated NaHCO_3_ aq., and the aqueous layer was reextracted with CH_2_Cl_2_. The organic layer was dried over Na_2_SO_4_ and was concentrated in vacuo. The crude was purified by silica gel column chromatography (CH_2_Cl_2_/MeOH = 10:1) to give **19** (0.57 g, 55%) as a purple solid.

The spectroscopic and physical data were identical to those reported [25].

#### 3.3.10. Ts-makaluvamine D (**20**)

Tyramine (37.9 mg, 0.28 mmol, 3.0 equiv.) was added to a solution of **19** (107.2 mg, 0.31 mmol) in dry MeOH/THF (1.6:1, 6.5 mL) at rt, and the mixture was stirred for 16 h. The reaction mixture was concentrated in vacuo, and the crude material was purified by silica gel column chromatography (10% MeOH/CH_2_Cl_2_, containing 0.1% TFA) to give **20** (88.2 mg, 49%) as a purple solid and recovered **19** (41.1 mg, 87% brsm).

The spectroscopic and physical data were identical to those reported [25].

#### 3.3.11. Makaluvamine D (**5**)

Pyridine hydrochloride (74.1 mg, 0.64 mmol, 10 equiv.) was added to a solution of **20** (36.9 mg, 64 µmol) in dry DMF (3 mL) at rt, and the mixture was stirred at 80 °C for 2 h. The reaction mixture was concentrated in vacuo, and the crude material was purified by silica gel column chromatography (10% MeOH/CH_2_Cl_2_, containing 0.1% TFA) to give **5** (18.9 mg, 70%) as a red-brown solid.

The spectroscopic and physical data were identical to those reported [13].

#### 3.3.12. Makaluvamine P (**10**)

NaH (50% *w*/*w*, 4.3 mg, 90 µmol, 2.0 equiv.) was added to a solution of **5** (18.9 mg, 45 µmol) in dry DMF (5 mL) at 0 °C, and the mixture was stirred for 2 h. Then, CH_3_I (0.81 mol/L in dry DMF, 0.17 mL, 0.13 mmol, 3.0 equiv.) was added dropwise to the reaction mixture, and the whole mixture was stirred for 2 h. The reaction mixture was quenched with MeOH and concentrated in vacuo. The crude material was purified by silica gel column chromatography (5–20% MeOH/CH_2_Cl_2_ gradient, containing 0.1% TFA) to give **10** (18.5 mg, 92%) as a red-brown solid.

The spectroscopic and physical data were identical to those reported [30].

#### 3.3.13. 5-Methyl-1-tosyl-1,3,4,5-tetrahydropyrrolo[4,3,2-*de*]quinoline-7,8-dione (Ts-damirone B, **22**)

K_2_CO_3_ (73.3 mg, 0.53 mmol, 2.0 equiv.) was added to a solution of **19** (90.8 mg, 0.26 mmol) in dry DMF (5 mL) at rt. Then, CH_3_I (1.61 M in dry DMF, 0.49 mL, 0.80 mmol, 3.0 equiv.) was added dropwise to the solution, and the whole mixture was stirred for 16 h. The reaction mixture was quenched with H_2_O and extracted with EtOAc four times. The organic layer was dried over Na_2_SO_4_ and was concentrated in vacuo. The crude material was purified by silica gel column chromatography (CH_2_Cl_2_/MeOH = 15:1) to give **22** (93.2 mg, quant.) as a red solid.

The spectroscopic and physical data were identical to those reported [31].

#### 3.3.14. Makaluvamine J (**1**)

Tyramine (37.9 mg, 0.28 mmol, 3.0 equiv.) was added to a solution of **22** (33.0 mg, 92.1 µmol) in dry MeOH/THF (1.6:1, 6.5 mL) at rt, and the whole mixture was stirred for 16 h. The reaction mixture was concentrated in vacuo, and the crude material was purified by silica gel column chromatography (5–20% MeOH/CH_2_Cl_2_ gradient, containing 0.1% TFA) to give **1** (14.8 mg, 37%) as a green solid and recovered **22** (16.4 mg, 87% brsm).

The spectroscopic and physical data were identical to those reported [13].

#### 3.3.15. 5-Methyl-8-oxo-7-(phenethylamino)-1,3,4,8-tetrahydropyrrolo[4,3,2-*de*]quinolin-5-ium trifluoroacetate (**23**)

Phenethylamine (0.79 M in dry MeOH/THF, 0.32 mL, 0.25 mmol, 3.0 equiv.) was added to a solution of **22** (30.0 mg, 84 µmol) in dry MeOH/THF (1.6:1, 6.5 mL) at rt, and the whole mixture was stirred for 16 h. The reaction mixture was concentrated in vacuo, and the crude material was purified by silica gel column chromatography (5–20% MeOH/CH_2_Cl_2_ gradient, containing 0.1% TFA) to give **23** (12.7 mg, 36%) as a green solid and recovered **22** (14.0 mg, 83% brsm).

^1^H NMR (500 MHz, CD_3_OD): δ 7.32–7.21 (m, 5H), 7.14 (s, 1H), 5.40 (s, 1H), 3.92 (t, *J* = 7.5 Hz, 2H), 3.72 (t, *J* = 7.0 Hz, 2H), 3.36 (s, 3H), 3.02 (t, *J* = 7.0 Hz, 2H), 3.00 (t, *J* = 7.5 Hz, 2H). ^13^C NMR (125 MHz, CD_3_OD): δ 168.1, 158.0, 155.2, 139.6, 130.1, 129.8, 127.8, 127.0, 124.9, 124.7, 120.0, 84.3, 54.4, 46.2, 39.7, 35.4, 20.3. MS (ESI-Q-TOF) *m/z*: 306 [M + H]^+^. HRMS (ESI-Q-TOF) *m/z*: 306.1606, calcd for C_19_H_20_N_3_O; found: 306.1607.

#### 3.3.16. 7-((2-(1*H*-Indol-3-yl)ethyl)amino)-5-methyl-8-oxo-1,3,4,8-tetrahydropyrrolo[4,3,2-*de*]quinolin-5-ium trifluoroacetate (**24**)

Tryptamine (36.9 mg, 0.23 mmol, 3.0 equiv.) was added to a solution of **22** (27.5 mg, 77 µmol) in dry MeOH/THF (1.6:1, 6.5 mL) at rt, and the whole mixture was stirred for 16 h. The reaction mixture was concentrated in vacuo, and the crude material was purified by silica gel column chromatography (5–20% MeOH/CH_2_Cl_2_ gradient, containing 0.1% TFA) to give **24** (7.7 mg, 22%) as a green solid and recovered **22** (15.2 mg, 74% brsm).

^1^H NMR (500 MHz, CD_3_OD): δ 7.61 (d, *J* = 7.8 Hz, 1H), 7.32 (d, *J* = 8.1 Hz, 1H), 7.08–6.99 (m, 4H) 4.96 (s, 1H), 3.76–3.73 (m, 4H), 3.14 (t, *J* = 6.3 Hz, 2H), 2.89 (t, *J* = 7.1 Hz, 2H), 2.88 (s, 3H). ^13^C NMR (125 MHz, CD_3_OD): δ 168.1, 157.4, 155.5, 138.3, 128.7, 127.0, 124.7, 124.5, 122.6, 120.0, 119.3, 119.1, 112.5, 112.3, 84.0, 54.1, 45.5, 39.2 25.9, 20.3. MS (ESI-Q-TOF) *m/z*: 345 [M + H]^+^. HRMS (ESI-Q-TOF) *m/z*: 345.1715, calcd for C_21_H_21_N_4_O; found: 345.1721.

#### 3.3.17. 1-Tosyl-5-vinyl-1,3,4,5-tetrahydropyrrolo[4,3,2-*de*]quinoline-7,8-dione (**25**)

K_2_CO_3_ (24.9 mg, 0.18 mmol, 2.0 equiv.) was added to a solution of **19** (30.8 mg, 90 µmol) in dry DMF (5 mL) at rt. Then, Allyl bromide (1.18 M in dry DMF, 0.23 mL, 0.27 mmol, 3.0 equiv.) was added dropwise to the solution, and the whole mixture was stirred for 16 h. The reaction mixture was quenched with H_2_O and extracted with EtOAc for four times. The organic layer was dried over Na_2_SO_4_ and was concentrated in vacuo. The crude material was purified by silica gel column chromatography (CH_2_Cl_2_/MeOH = 30:1) to give **25** (31.9 mg, 93%) as a red solid.

^1^H NMR (500 MHz, CDCl_3_): δ 8.11 (d, *J* = 8.5 Hz, 2H), 7.54 (s, 1H), 7.32 (d, *J* = 8.5 Hz, 2H), 5.82–5.75 (m, 1H), 5.38 (s, 1H), 5.30–5.23 (m, 2H), 3.94 (d, *J* = 5.6 Hz, 2H), 3.57 (d, *J* = 6.7 Hz, 2H), 2.90 (d, *J* = 6.7 Hz, 2H), 2.41 (s, 3H). ^13^C NMR (125 MHz, CDCl_3_): δ 177.8, 168.5, 151.6, 146.4, 133.6, 130.1, 130.0, 129.9, 129.4, 125.7, 124.5, 119.3, 116.7, 95.3, 54.0, 49.0, 21.9, 20.2. MS (ESI-Q-TOF) *m/z*: 383 [M + H]^+^. HRMS (ESI-Q-TOF) *m/z*: 383.1066, calcd for C_20_H_19_N_2_O_4_S; found: 383.1063.

#### 3.3.18. 5-Benzyl-1-tosyl-1,3,4,5-tetrahydropyrrolo[4,3,2-*de*]quinoline-7,8-dione (**26**)

K_2_CO_3_ (15.0 mg, 0.11 mmol, 2.0 equiv.) was added to a solution of **19** (18.6 mg, 54 µmol) in dry DMF (5 mL) at rt. Benzyl bromide (0.84 M in dry DMF, 0.19 mL, 0.16 mmol, 3.0 equiv.) was added dropwise to a solution, and the mixture was stirred for 16 h. The reaction mixture was quenched with H_2_O and extracted with EtOAc four times. The organic layer was dried over Na_2_SO_4_ and was concentrated in vacuo. The crude material was purified by silica gel column chromatography (CH_2_Cl_2_/MeOH = 30:1) to give **26** (18.2 mg, 77%) as a purple solid.

^1^H NMR (500 MHz, CDCl_3_): δ 8.12 (d, *J* = 8.5 Hz, 2H), 7.55 (s, 1H), 7.36–7.30 (m, 5H), 7.23 (d, *J* = 6.6 Hz, 2H), 5.51 (s, 1H), 4.52 (s, 2H), 3.58 (t, *J* = 6.7 Hz, 2H), 2.87 (t, *J* = 6.7 Hz, 2H), 2.41 (s, 3H). ^13^C NMR (125 MHz, CDCl_3_): δ 177.8, 168.5, 152.2, 146.5, 134.9, 133.6, 130.0, 129.9, 129.5, 129.2, 128.3, 127.5, 125.8, 124.7, 116.8, 95.3, 54.8, 49.1, 21.9, 20.2. MS (ESI-Q-TOF) *m/z*: 433 [M + H]^+^. HRMS (ESI-Q-TOF) *m/z*: 433.1222, calcd for C_24_H_21_N_2_O_4_S; found: 433.1201.

#### 3.3.19. 5-Allyl-7-((4-hydroxyphenethyl)amino)-8-oxo-1,3,4,8-tetrahydropyrrolo[4,3,2-*de*]quinolin-5-ium trifluoroacetate (**27**)

Tyramine (29.9 mg, 0.22 mmol, 3.0 equiv.) was added to a solution of **25** (27.8 mg, 72.7 µmol) in dry MeOH/THF (1.6:1, 6.5 mL) at rt, and the whole mixture was stirred for 16 h. The reaction mixture was concentrated in vacuo, and the crude material was purified by silica gel column chromatography (5–20% MeOH/CH_2_Cl_2_ gradient, containing 0.1% TFA) to give **27** (7.5 mg, 22%) as a green solid and recovered **25** (18.5 mg, 89% brsm).

^1^H NMR (500 MHz, CD_3_OD): δ 7.15 (s, 1H), 7.06 (d, *J* = 8.5 Hz, 2H), 6.71 (d, *J* = 8.5 Hz, 2H), 5.97–5.90 (m, 1H), 5.41–5.36 (m, 3H), 4.32 (d, *J* = 5.7 Hz, 2H), 3.91 (t, *J* = 7.5 Hz, 2H), 3.65 (t, *J* = 6.9 Hz, 2H), 2.99 (t, *J* = 7.5 Hz, 2H), 2.89 (t, *J* = 6.9 Hz, 2H). ^13^C NMR (125 MHz, CD_3_OD): δ 168.0, 158.0, 157.5, 155.6, 131.6, 131.0, 130.1, 127.1, 125.1, 124.9, 119.9, 119.4, 116.5, 84.5, 55.2, 52.5, 46.6, 34.8, 20.5. MS (ESI-Q-TOF) *m/z*: 348 [M + H]^+^. HRMS (ESI-Q-TOF) *m/z*: 348.1712, calcd for C_21_H_22_N_3_O_2_; found: 348.1729.

#### 3.3.20. 5-Benzyl-7-((4-hydroxyphenethyl)amino)-8-oxo-1,3,4,8-tetrahydropyrrolo[4,3,2-*de*]quinolin-5-ium trifluoroacetate (**28**)

Tyramine (27.9 mg, 0.20 mmol, 3.0 equiv.) was added to a solution of **26** (29.3 mg, 68 µmol) in dry MeOH/THF (1.6:1, 6.5 mL) at rt, and the whole mixture was stirred for 16 h. The reaction mixture was concentrated in vacuo, and the crude material was purified by silica gel column chromatography (5–20% MeOH/CH_2_Cl_2_ gradient, containing 0.1% TFA) to give **28** (5.8 mg, 17%) as a green solid and recovered **26** (17.3 mg, 76% brsm).

^1^H NMR (500 MHz, CD_3_OD): δ 7.44 (t, *J* = 7.3 Hz, 2H), 7.37(m, 3H), 7.16 (s, 1H), 7.00 (d, *J* = 8.5 Hz, 2H), 6.69 (d, *J* = 8.5 Hz, 2H), 5.57 (s, 1H), 4.93 (s, 2H), 3.91 (t, *J* = 7.5 Hz, 2H), 3.62 (t, *J* = 7.0 Hz, 2H), 2.95 (t, *J* = 7.5 Hz, 2H), 2.83 (t, *J* = 7.0 Hz, 2H). ^13^C NMR (125 MHz, CD_3_OD): δ 168.0, 158.3, 157.5, 155.8, 136.0, 131.0, 130.4, 130.0, 129.6, 128.7, 127.2, 125.2, 124.9, 119.9, 116.5, 84.8, 56.1, 52.7, 46.6, 34.7, 20.5. MS (ESI-Q-TOF) *m/z*: 398 [M + H]^+^. HRMS (ESI-Q-TOF) *m/z*: 398.1869, calcd for C_25_H_24_N_3_O_2_; found: 398.1884.

## Figures and Tables

**Table 1:** Cytotoxicity of makaluvamine J (**1**) and analogs against cancer cells.

Compound	PANC-1 (µM) ^1^	KB3-1 (µM) ^1^	S.I. ^2^
**1**	0.046	0.2	4.3
**5**	4.4	3.2	0.7
**10**	0.2	0.51	2.6
**23**	0.042	0.41	9.8
**24**	0.029	0.38	13.1
**25**	2.9	>10	>3.4
**26**	1.9	6.7	3.5
**27**	0.038	0.078	2.1
**28**	0.027	0.047	1.7

^1^ IC_50_ against respective cells. ^2^ Selective index: IC_50_ against KB3-1 cells/IC_50_ against PANC-1 cells.

## Data Availability

Data are contained within the article and Supplementary Materials.

## Supplementary Materials

The following supporting information can be downloaded at: https://www.mdpi.com/article/10.3390/molecules29061389/s1, Supplementary Data S1: The NMR spectra of new compounds.
