# Supplementary material for: Unified Synthesis and Biological Evaluation of Makaluvamine J and Its Analogs
Source: Molecules. 2024 Mar 20;29(6):1389. doi: 10.3390/molecules29061389 (PMC10976149; doi:10.3390/molecules29061389)

Supporting Information

Unified Synthesis and Biological Evaluation of Makaluvamine J and its Analogs

Yo Kiichi, Koshiro Fukuoka, Anna Kitano, Koya Ishino, Naoyuki Kotoku*

College of Pharmaceutical Sciences, Ritsumeikan University, 1-1-1 Noji-higashi, Kusatsu, Shiga, 525-8577, Japan

Contents

^{1}H- and ^{13}C-NMR spectra for new compounds: pages S2-S11

^{1}H NMR (500 MHz, $CDCl_3$)

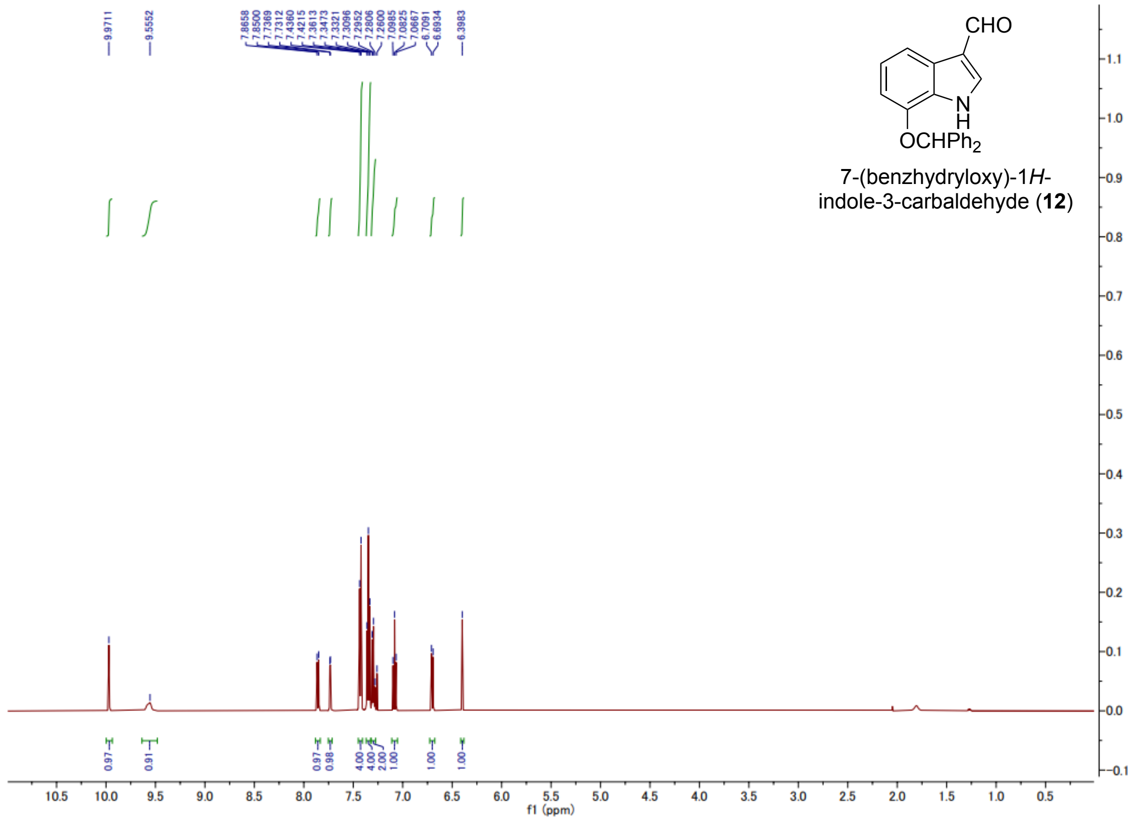

7-(benzhydryloxy)-1*H*-indole-3-carbaldehyde (**12**)

^{13}C NMR (125 MHz, $CDCl_3$)

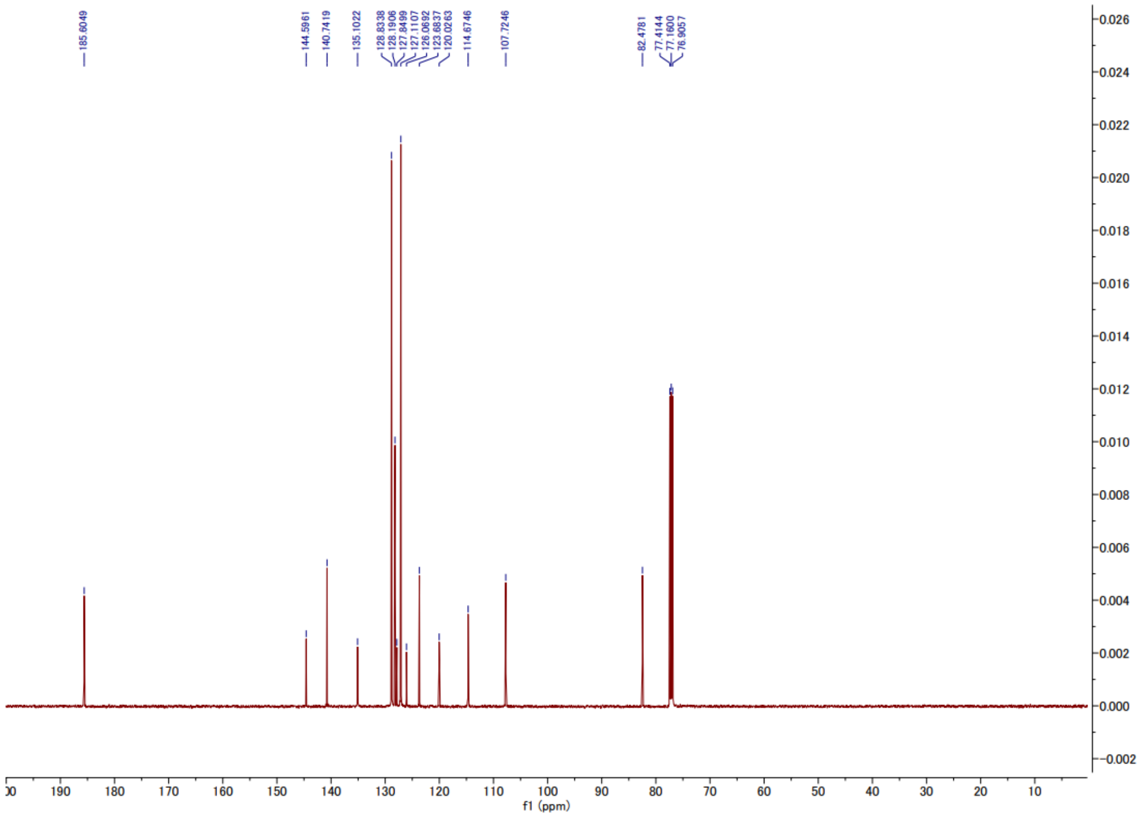

[1]**H NMR** (500 MHz, $(CD_3)_2CO$)

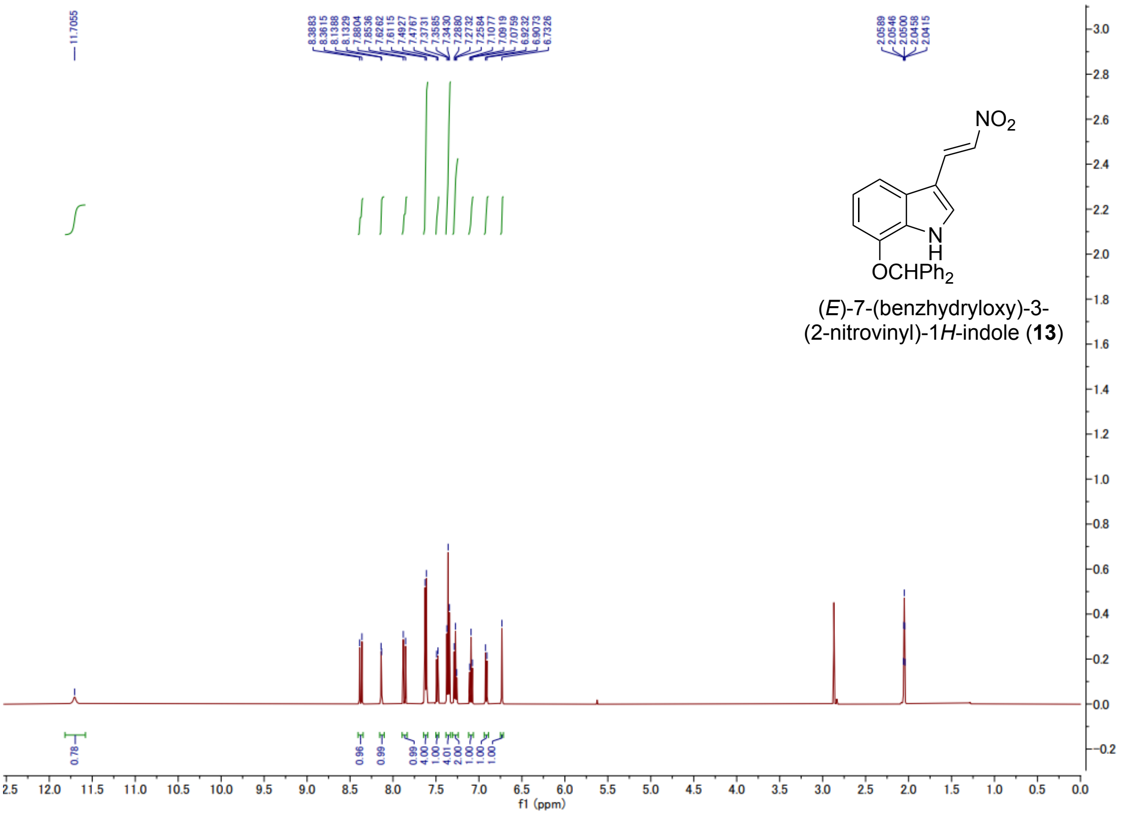

[13]**C NMR** (125 MHz, $(CD_3)_2CO$)

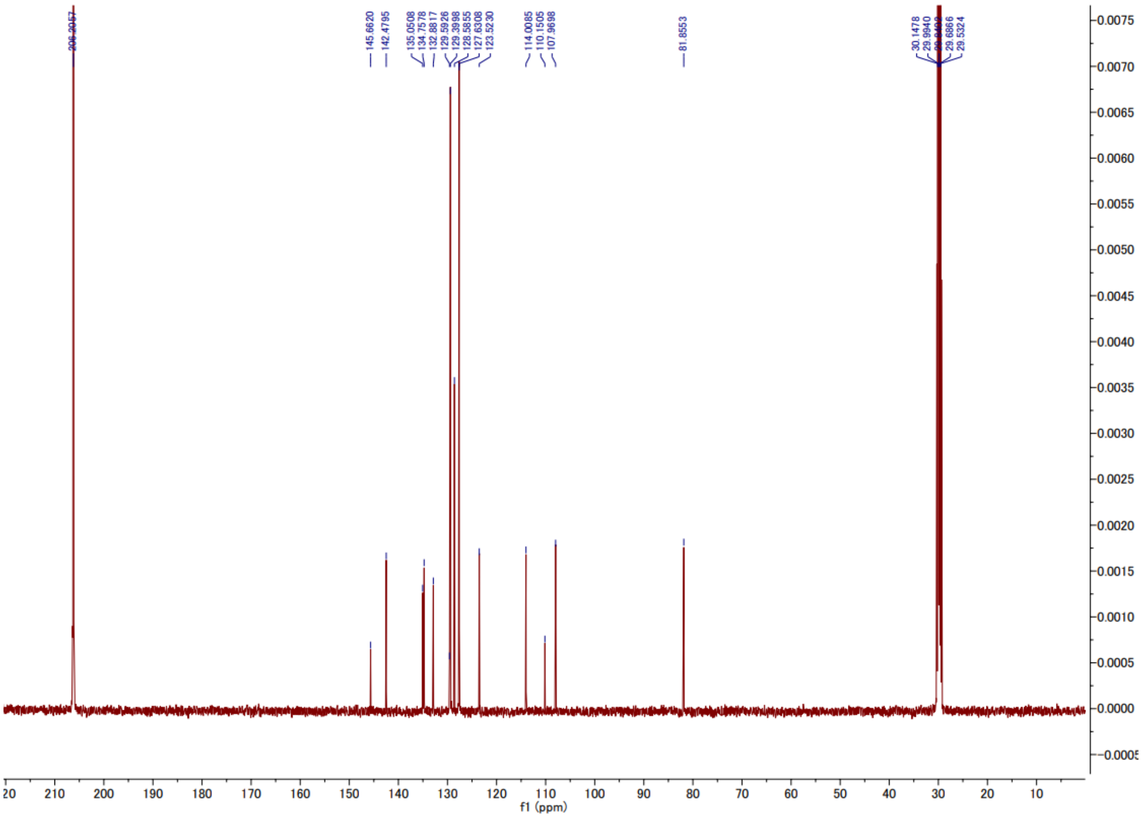

^{1}H NMR (500 MHz, $CDCl_3$)

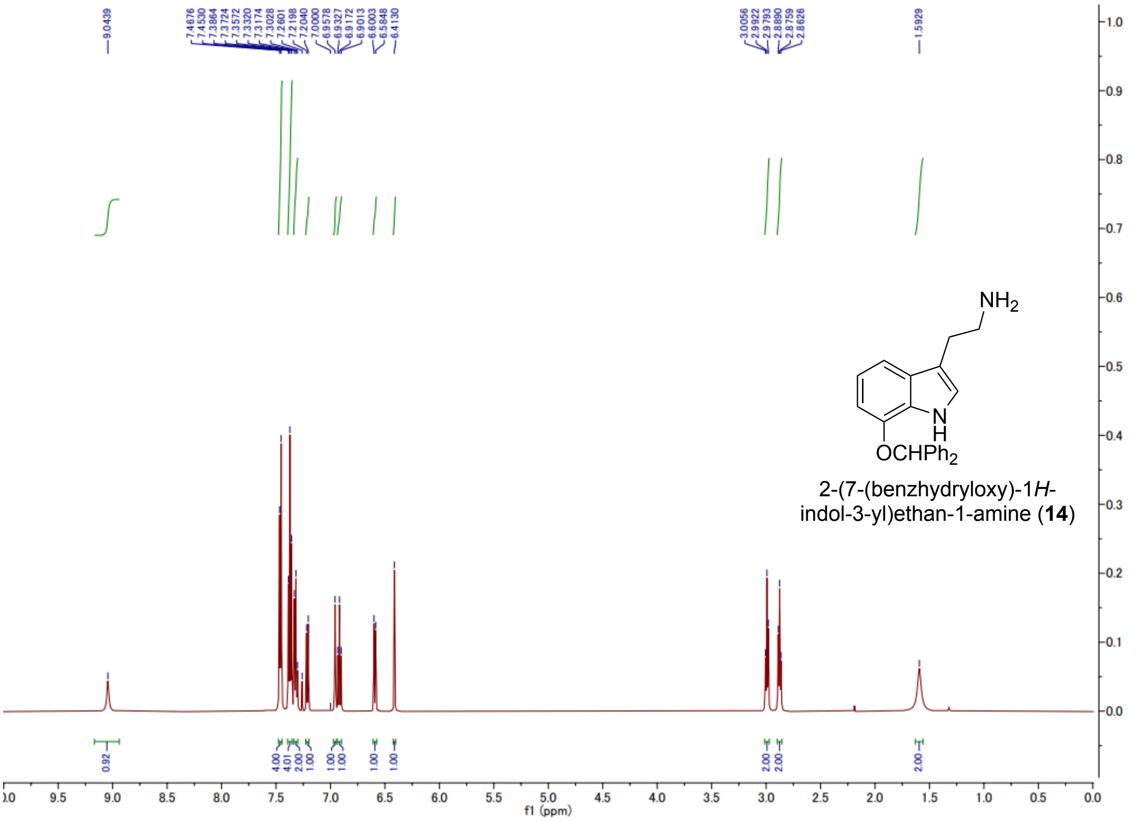

2-(7-(benzhydryloxy)-1*H*-indol-3-yl)ethan-1-amine (**14**)

^{13}C NMR (125 MHz, $CDCl_3$)

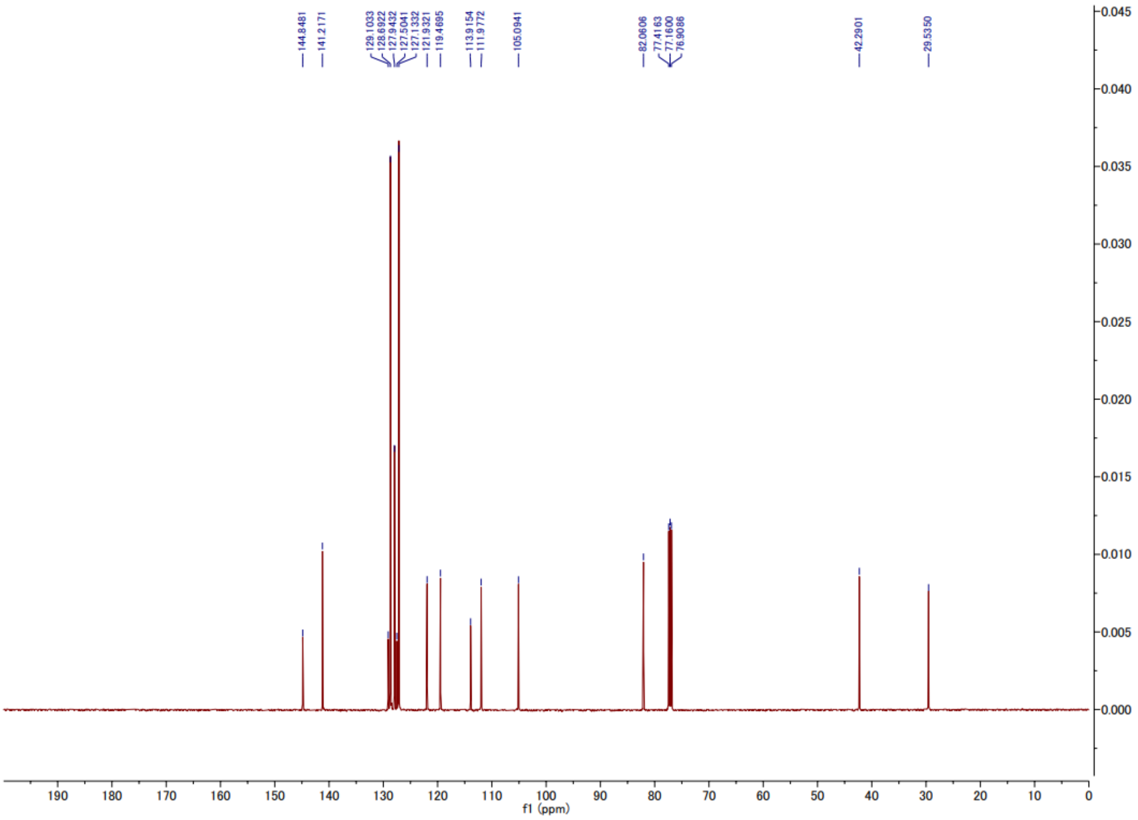

^{1}H NMR (500 MHz, $CDCl_3$)

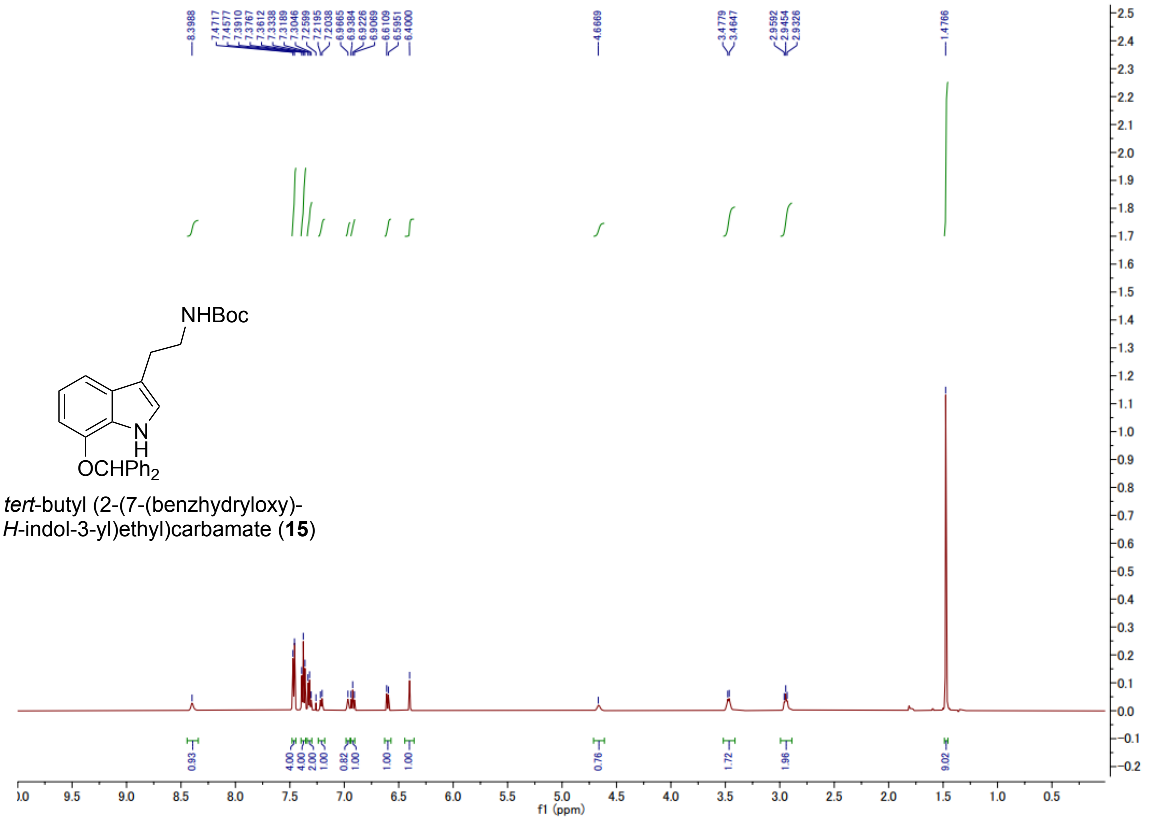

tert-butyl (2-(7-(benzhydryloxy)-1*H*-indol-3-yl)ethyl)carbamate (**15**)

^{13}C NMR (125 MHz, $CDCl_3$)

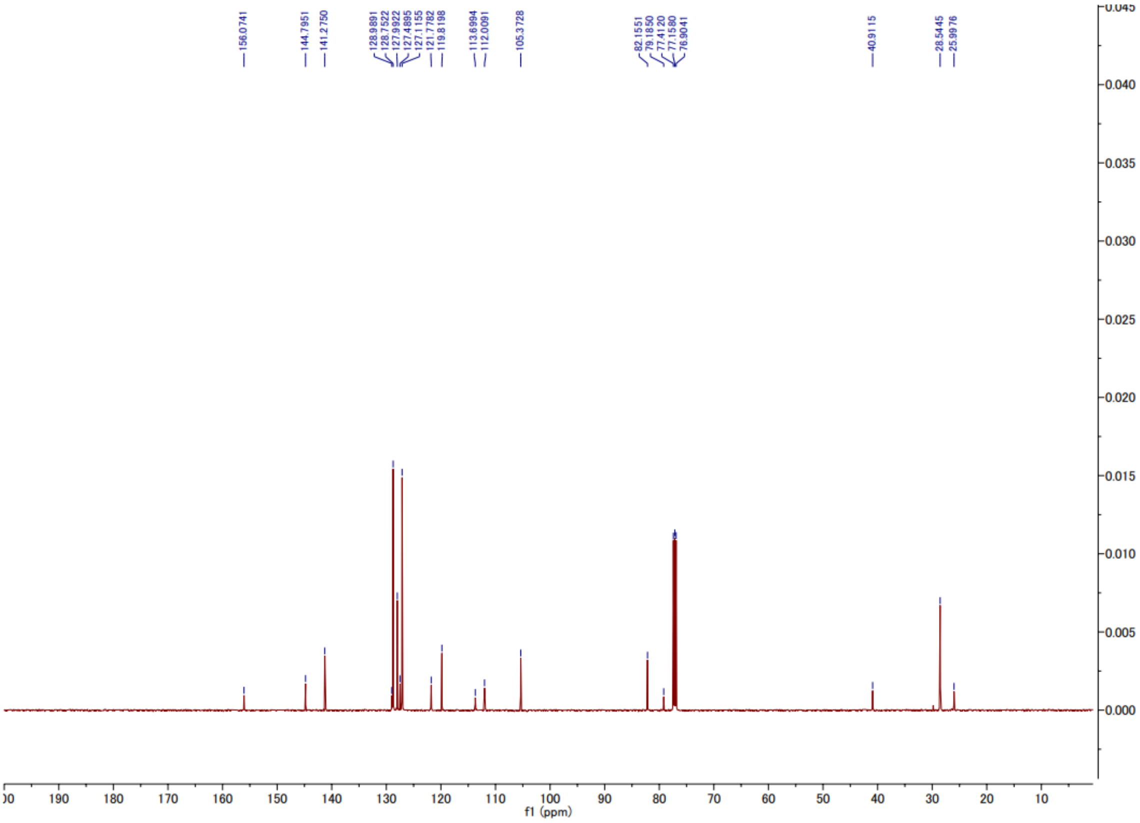

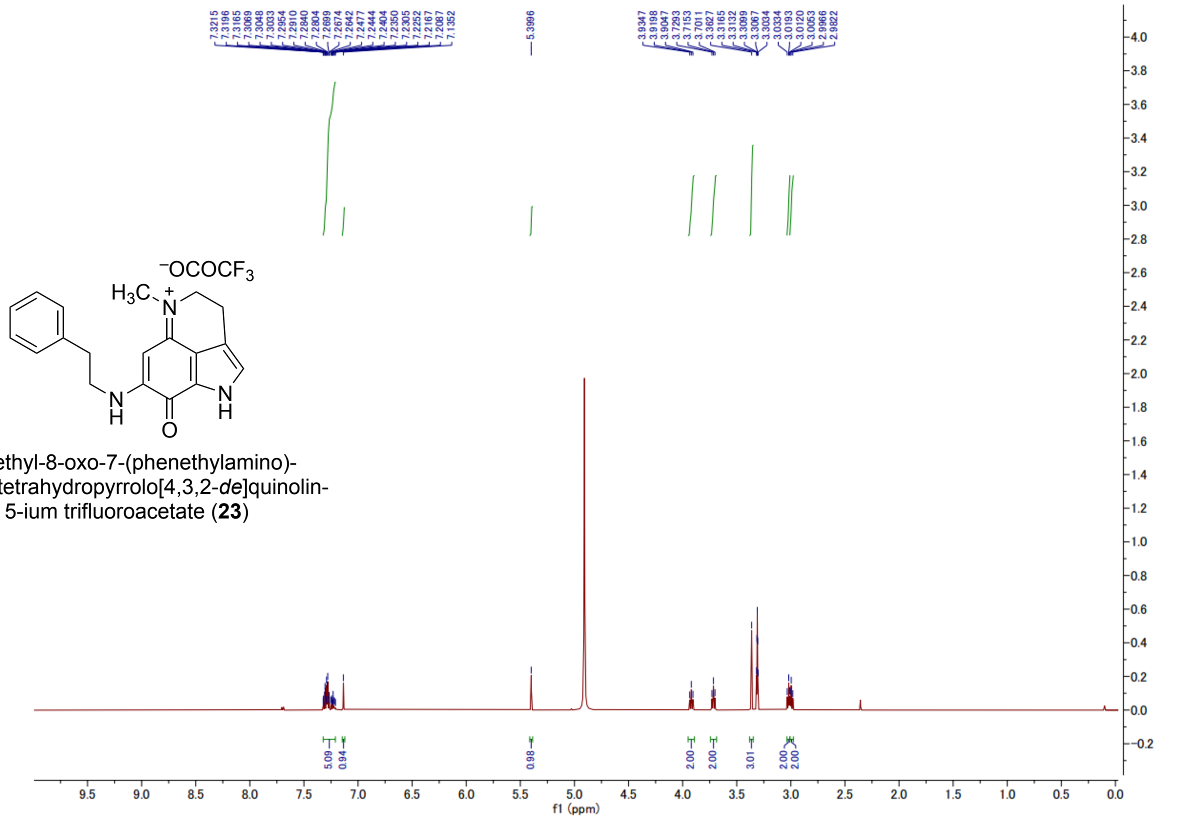

5-methyl-8-oxo-7-(phenethylamino)-1,3,4,8-tetrahydropyrrolo[4,3,2-*de*]quinolin-5-ium trifluoroacetate (**23**)

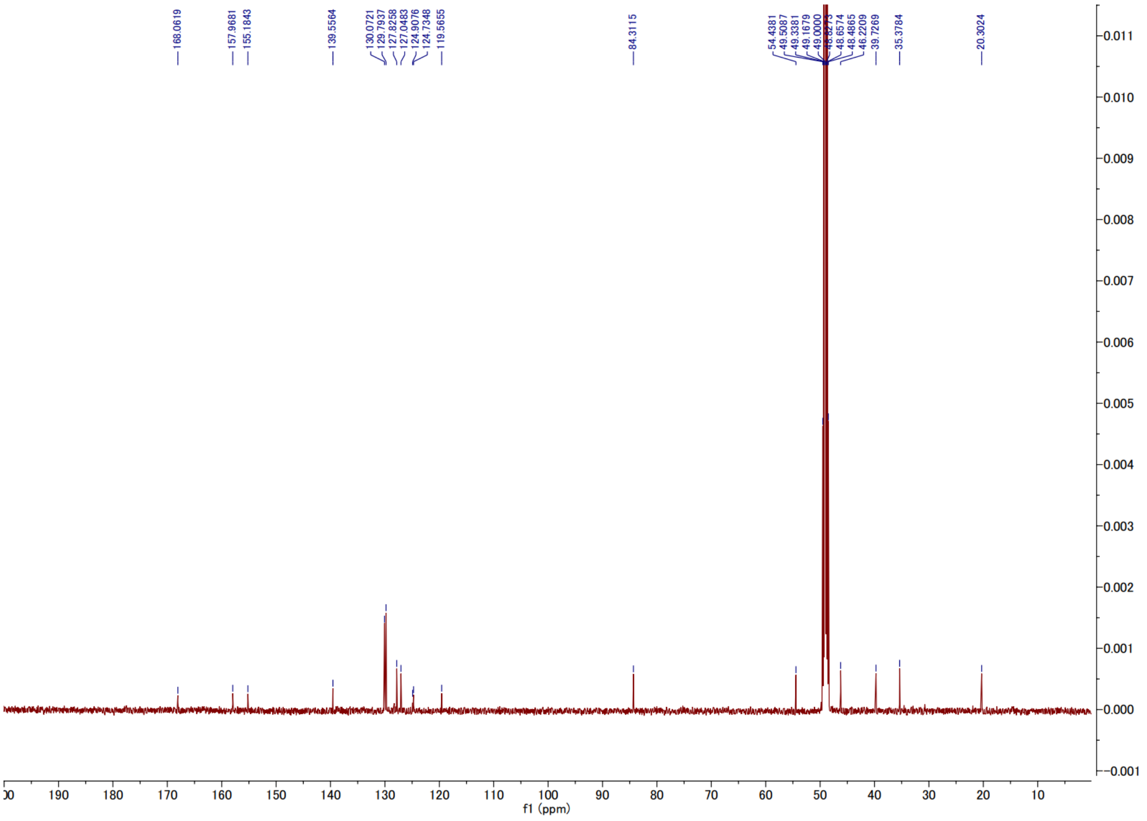

^{1}H NMR (500 MHz, CD_3OD)

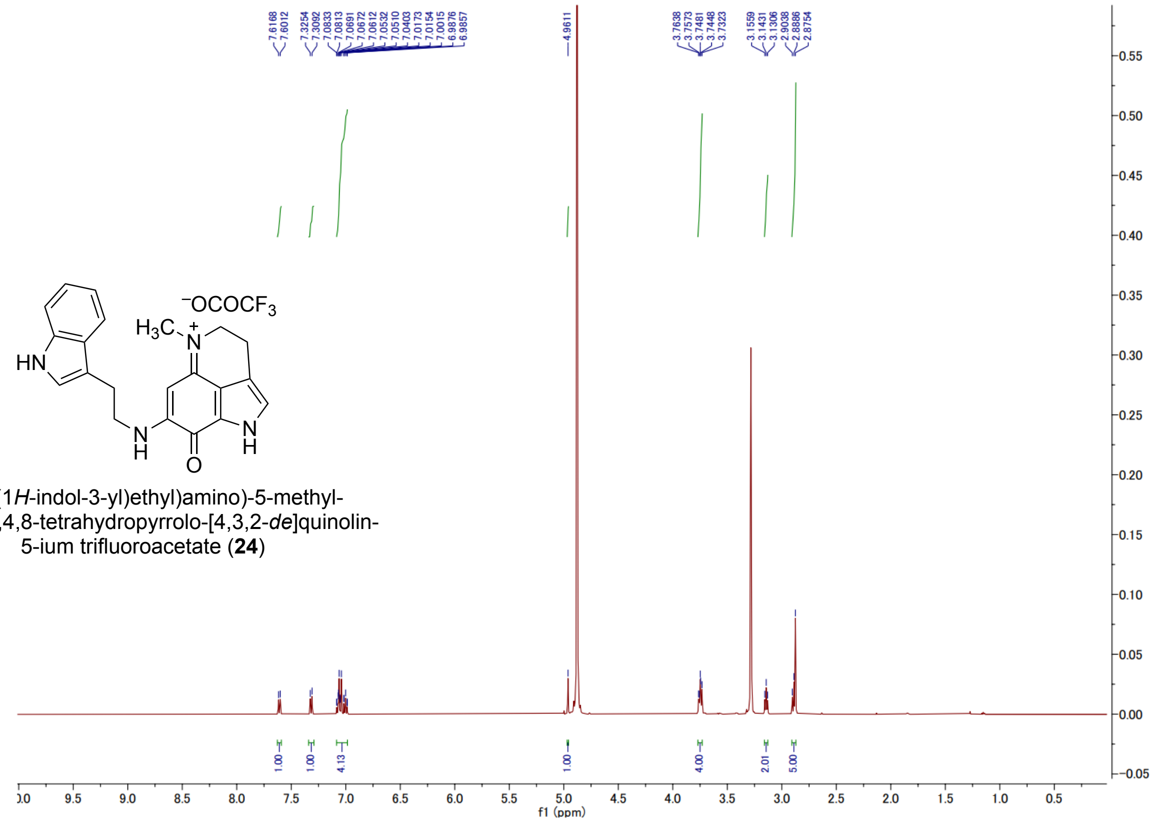

7-((2-(1*H*-indol-3-yl)ethyl)amino)-5-methyl-8-oxo-1,3,4,8-tetrahydropyrrolo-[4,3,2-*de*]quinolin-5-ium trifluoroacetate (**24**)

^{13}C NMR (125 MHz, CD_3OD)

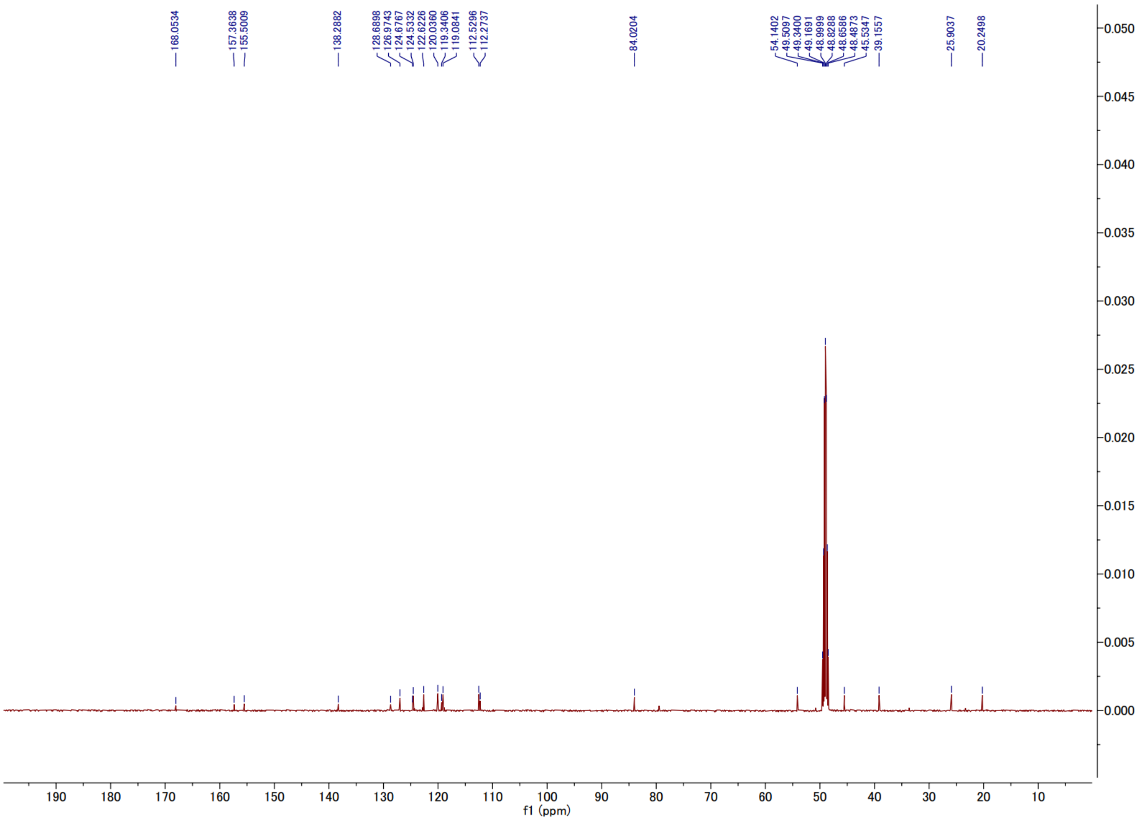

^{1}**H NMR** (500 MHz, $CDCl_3$)

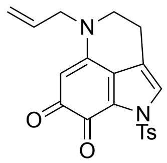

5-allyl-1-tosyl-1,3,4,5-tetrahydropyrrolo[4,3,2-*de*]quinoline-7,8-dione (**25**)

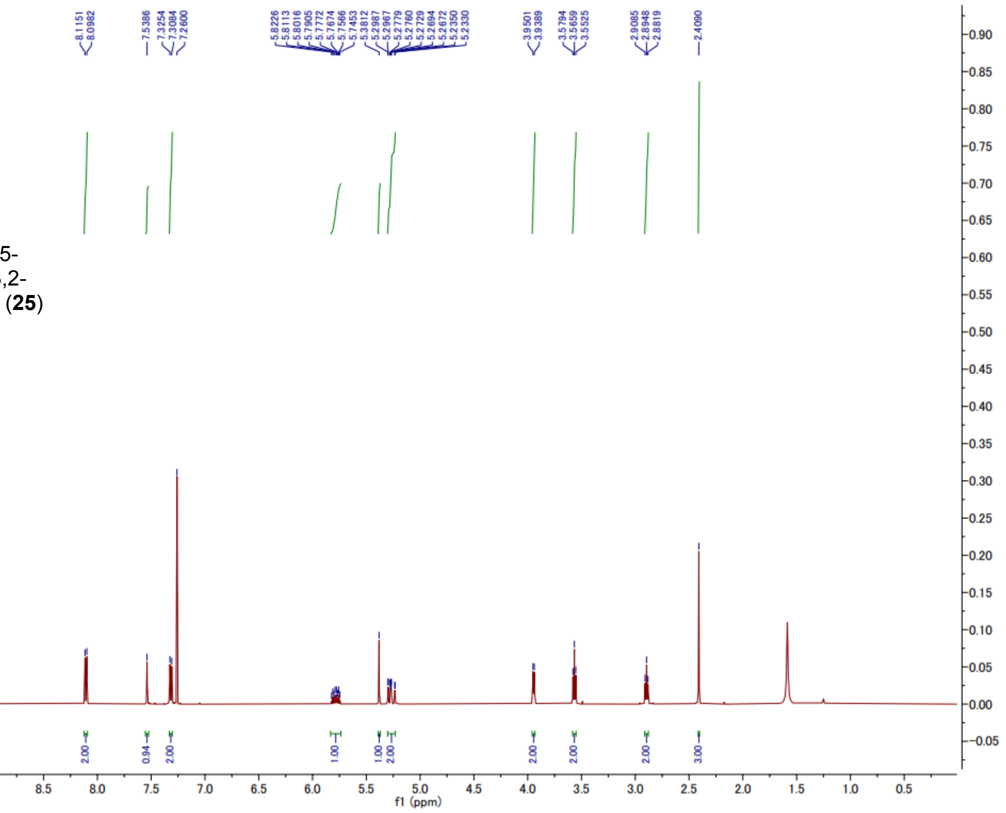

^{13}**C NMR** (125 MHz, $CDCl_3$)

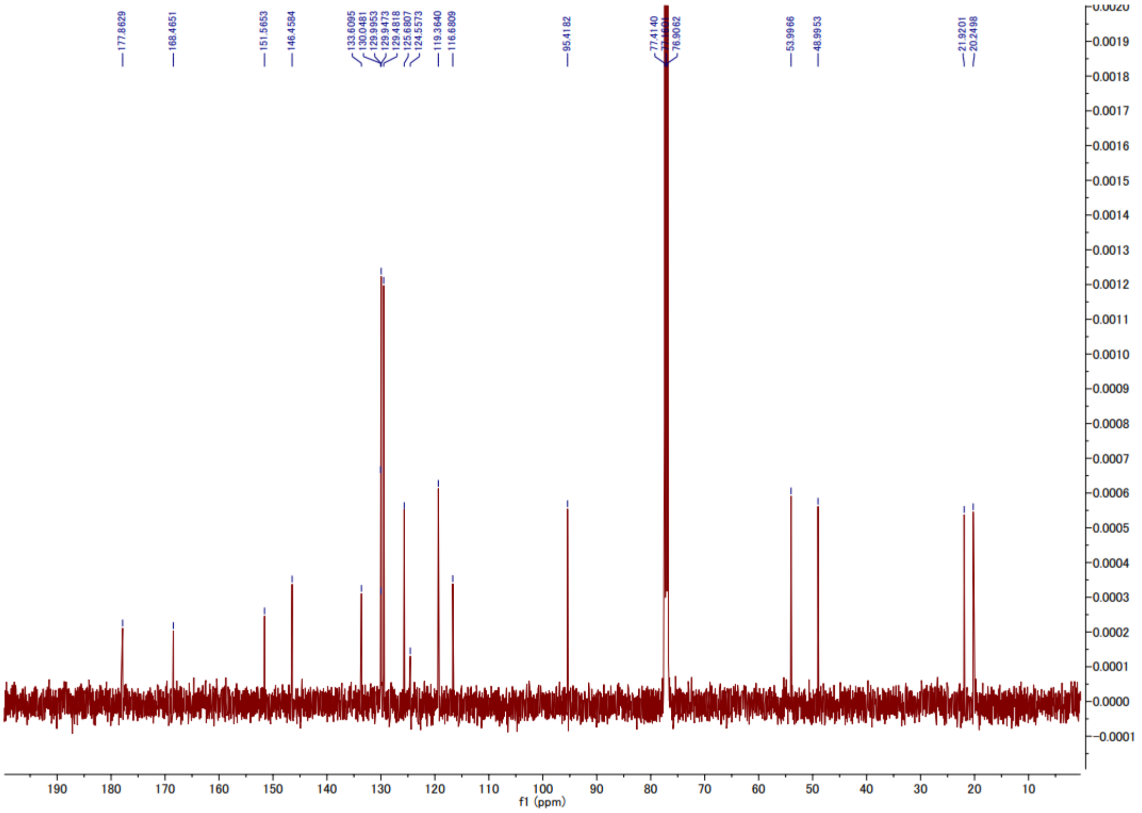

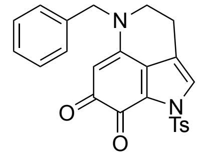

5-benzyl-1-tosyl-1,3,4,5-tetrahydropyrrolo[4,3,2-*de*]quinoline-7,8-dione (**26**)

^{1}H NMR (500 MHz, $CDCl_3$)

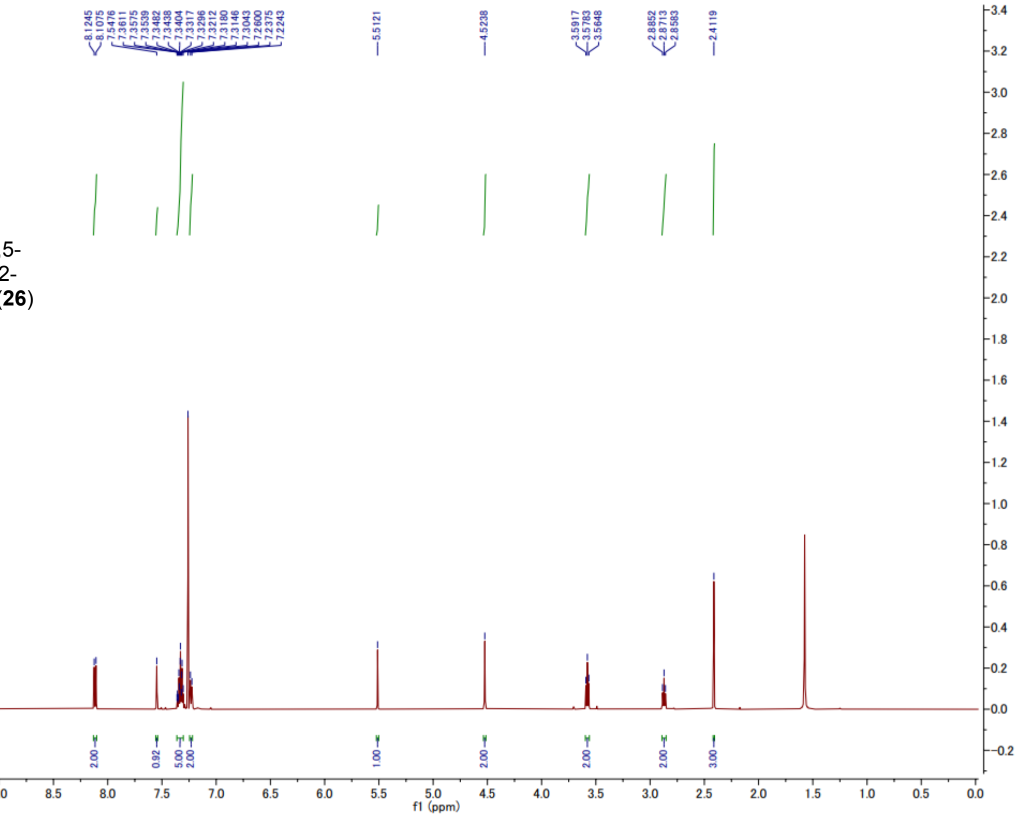

^{13}C NMR (125 MHz, $CDCl_3$)

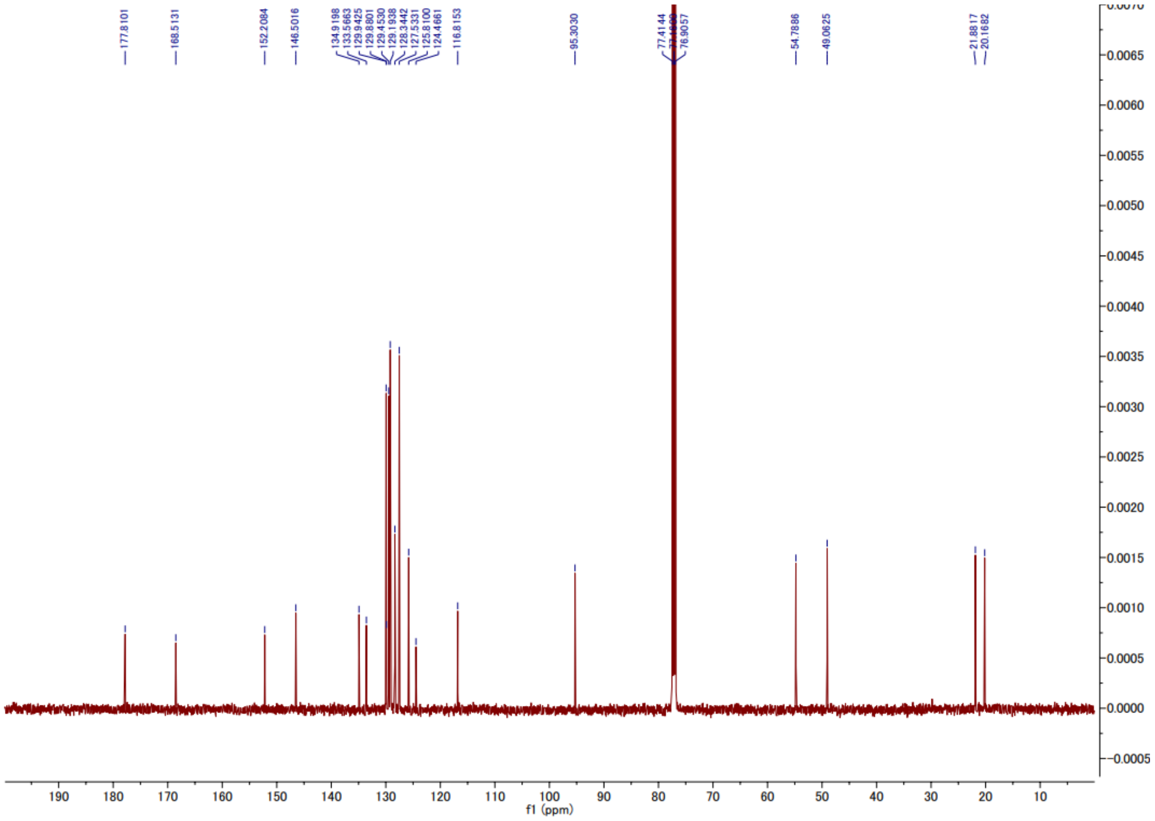

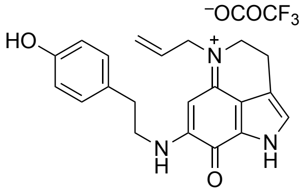

5-allyl-7-((4-hydroxyphenethyl)amino)-8-oxo-1,3,4,8-tetrahydropyrrolo[4,3,2-*de*]quinolin-5-ium trifluoroacetate (**27**)

^{1}H NMR (500 MHz, CD_3OD)

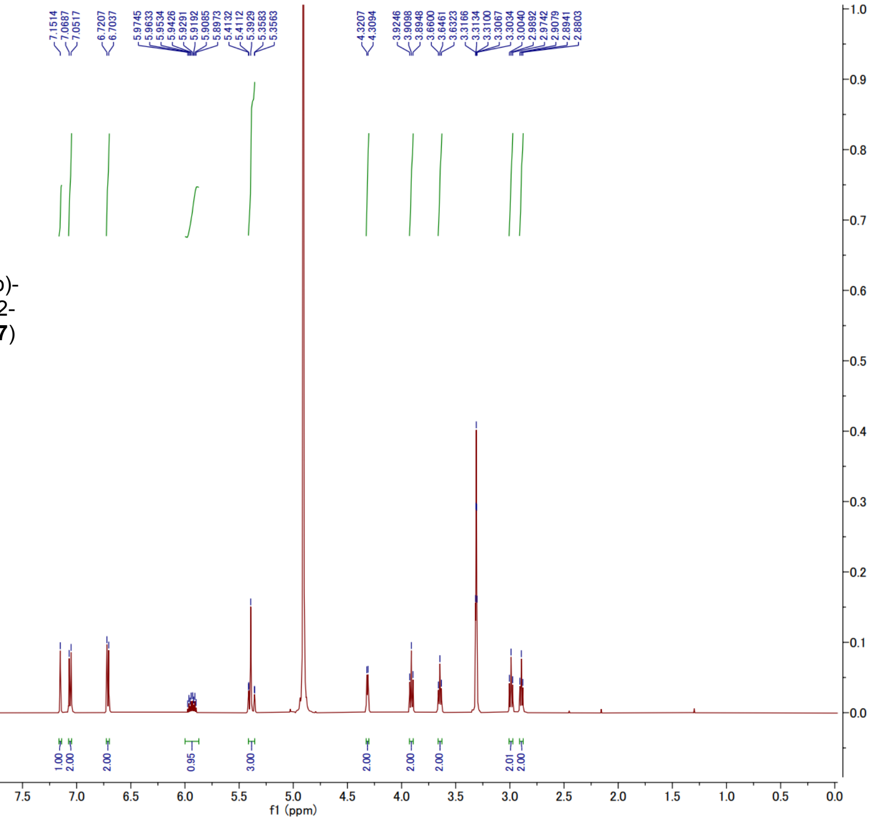

^{13}C NMR (125 MHz, CD_3OD)

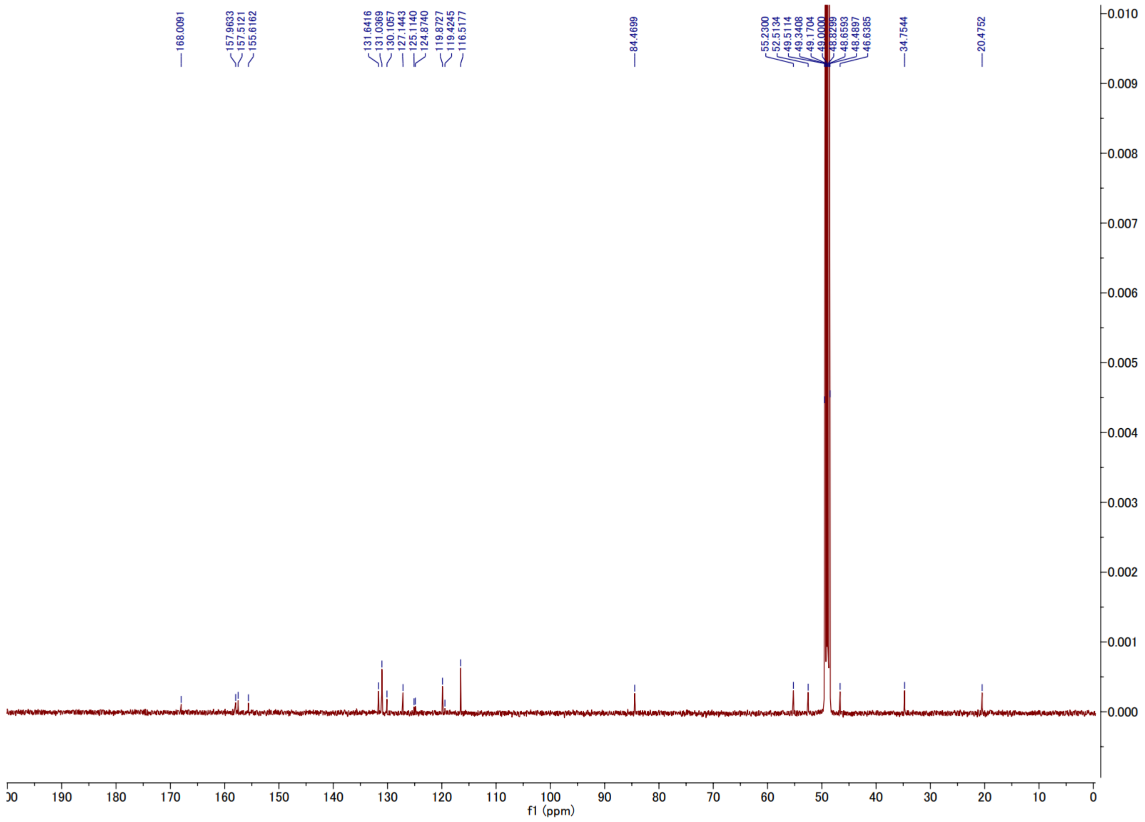

^{1}H NMR (500 MHz, CD_3OD)

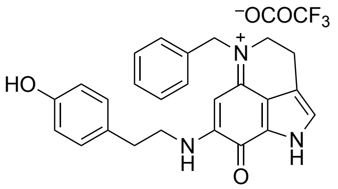

5-benzyl-7-((4-hydroxyphenethyl)amino)-8-oxo-1,3,4,8-tetrahydropyrrolo[4,3,2-*de*]quinolin-5-ium trifluoroacetate (**28**)

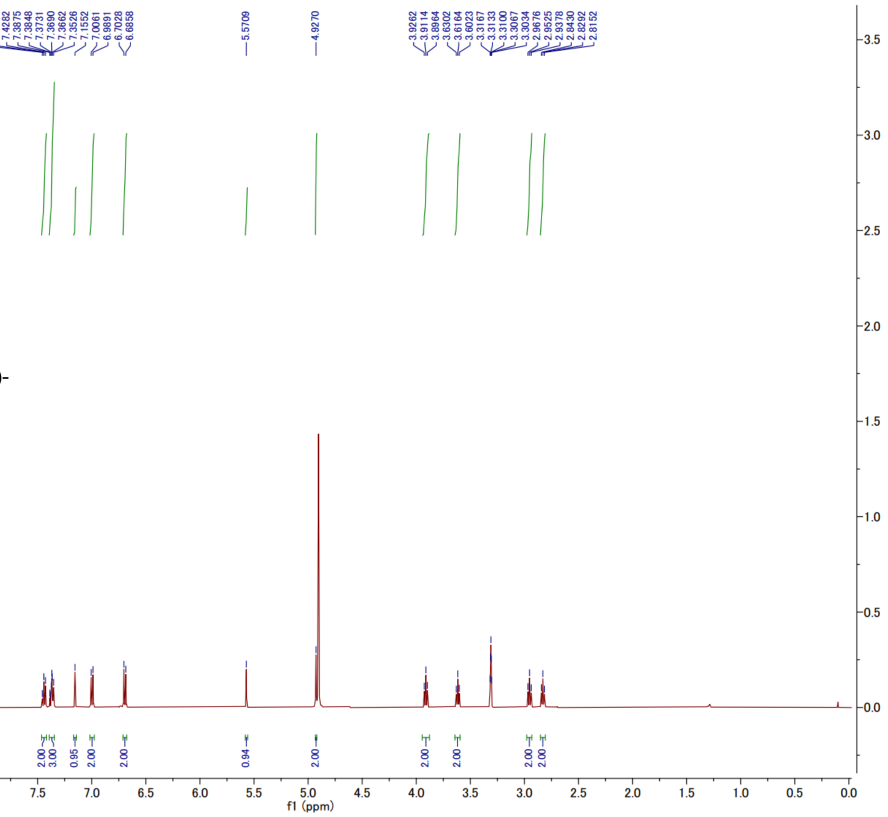

^{13}C NMR (125 MHz, CD_3OD)

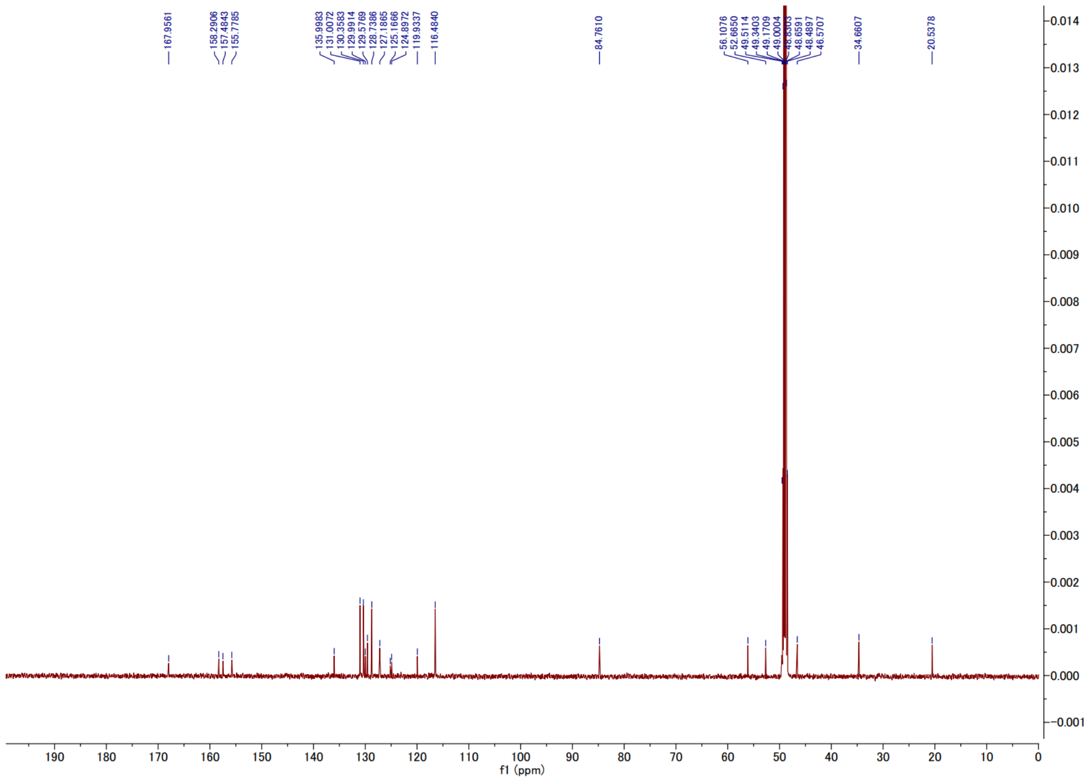

Supplement: Supplementary file 1 [file molecules-29-01389-s001.zip › molecules-2886988-supplementary.pdf]
